# Research progress on glioma drug resistance: mechanism analysis and therapeutic strategies

**DOI:** 10.3389/fphar.2026.1928598

**Published:** 2026-08-21

**Authors:** Zetong Bai, Haiguang Liu, Yan Wang, Haipeng Liu, Haipeng Xie, Hongan Fei, Xichao Wen, Wensong Wu, Mengye Li, Kebin Zheng

**Affiliations:** Department of Neurosurgery, Affiliated Hospital of Hebei University, Baoding, China

**Keywords:** drug resistance, glioma, hippo pathway, mitochondrial function, multidrug resistance transporter protein, nano drug delivery system, non coding RNA, tumor microenvironment

## Abstract

Glioma, especially glioblastoma multiforme (GBM), has become one of the major clinical challenges in the field of neurooncology due to its high invasiveness and significant multidrug resistance (MDR). The mechanism of drug resistance is complex and involves the synergy of many factors, including the overexpression of MDR related transporters such as P-glycoprotein (P-gp), multidrug resistance associated protein 1 (MRP1) and breast cancer resistance protein (BCRP), the characteristics of tumor stem cells, and the influence of tumor microenvironment (especially hypoxia). In addition, abnormally activated signaling pathways (such as Hippo pathway), abnormal regulation of non coding RNA and mitochondrial dysfunction also contribute to treatment failure. This article reviews the molecular research progress in the mechanism of drug resistance in glioma in recent years, focusing on the dynamic regulation of MDR protein, the drug resistance mediated by tumor stem cells, the impact of tumor microenvironment on drug efficacy, and the role of signaling pathways and non coding RNA in the development of drug resistance. At the same time, this paper also discusses the emerging strategies of drug delivery system and combination therapy based on nanotechnology. Through the systematic integration of existing studies, the aim is to deeply analyze the mechanism of drug resistance, provide theoretical basis and direction for the development of more effective treatment, and ultimately help improve the clinical prognosis.

## 1 Introduction

Gliomas are the most common malignant tumors in the central nervous system, and glioblastoma multiforme (GBM) is the most aggressive and lethal subtype. Despite the continuous progress of surgery, radiotherapy and chemotherapy, the prognosis of patients with GBM is generally poor, and the median survival time is usually only 12–15 months (Luiz et al., 2021; Bhusare and Kumar, 2024). The main reasons are that the tumor is highly invasive, the blood-brain barrier hinders drug delivery, and significant multidrug resistance (MDR)—which seriously weakens the efficacy of chemotherapy drugs represented by temozolomide (TMZ) (Pu et al., 2025; Sahoo et al., 2025). Therefore, in-depth analysis of the molecular mechanism of glioma drug resistance and the development of new treatment strategies to overcome drug resistance are of great significance to improve the survival and quality of life of patients.

The formation of drug resistance in glioma stems from the complex interaction between the internal characteristics of tumor cells and the external microenvironment, which is driven by multiple factors. One of the key mechanisms is the overexpression of ATP binding cassette (ABC) transporter family members, such as P-glycoprotein (MDR1/ABCB1), breast cancer resistance protein (BCRP/ABCG2) and multidrug resistance associated proteins (MRPs). These proteins can rely on ATP hydrolysis to actively excrete chemotherapeutic drugs to the outside of the cell, significantly reducing the accumulation concentration of drugs in the cell, thus weakening its therapeutic effect (Fan et al., 2023a; You et al., 2020; Shen et al., 2025). The expression and function of these transporters are regulated by epigenetic modifications and non coding RNAs [such as microRNA and long non coding RNA (*lncRNA*)], thus jointly promoting the formation of multidrug resistance phenotypes (Wang et al., 2021a; Li and Gao, 2021). In addition, glioma stem cells (GSCs), a subset of cells with the ability of self-renewal and tumor initiation, are considered to be the key factors driving treatment resistance and tumor recurrence (Batara et al., 2023; Nasrolahi et al., 2023). At the same time, the tumor microenvironment further exacerbates the development of drug resistance by activating survival promoting signaling pathways and blocking effective drug delivery (Liu and Yu, 2025).

At the molecular level, there are several key pathways in drug-resistant gliomas. Abnormal activation of DNA repair mechanisms, especially those involving O6 methylguanine DNA methyltransferase (*MGMT*), mismatch repair (MMR) and base excision repair (BER), enables tumor cells to resist DNA damage induced by alkylating agents such as TMZ (Pu et al., 2025; Gu et al., 2025). PI3K/Akt/mtor, notch, wnt/β-catenin, hedgehog and Hippo signaling pathways are also involved in promoting cell survival, proliferation and dryness, thereby enhancing drug resistance (Wu H. et al., 2022). Non coding RNAs (including long non coding RNAs such as *EPIC1* and circular RNA) have been identified as key regulators of these pathways and drug resistance mechanisms (Wang J. et al., 2020; Mafi et al., 2024). In addition, hypoxia microenvironment can induce the expression of HIF-1 α, HIF-2 α and other factors, which upregulate IGF1R and other survival and drug resistance genes, further complicating the treatment response (Liao B. et al., 2025).

How to break through the existing treatment bottleneck has become the research focus in this field, and also promoted the emergence of a variety of innovative treatment schemes. The integration of multiomics analysis and single-cell transcriptomics is providing more in-depth insights into tumor heterogeneity, drug resistance mechanism and spatial cell structure, thus promoting the discovery of new biomarkers and individualized therapeutic targets (Gu et al., 2025; Zheng et al., 2023). Drug delivery systems based on nanotechnology have shown great potential in improving the ability of drugs to penetrate the blood-brain barrier (BBB), optimizing tumor targeting, and realizing the coordinated delivery of chemotherapeutic drugs and drug resistance regulators (Xu et al., 2021a; Sandbhor et al., 2023). For example, polymer nanocarriers and exosomes simulation systems have been developed for efficient delivery of temozolomide, docetaxel and other drugs, and can simultaneously load active ingredients that regulate tumor microenvironment or inhibit drug resistance pathways (Rehman et al., 2022; Liu et al., 2024). At the same time, the research on molecular inhibitors targeting the glioma stem cell microenvironment and its related signaling pathways (such as Gli, notch, hippo) is also actively promoted (Zhang et al., 2022; D’amico and De amicis, 2022; Casati et al., 2021). The active ingredients of natural compounds represented by curcumin can effectively reverse drug resistance and enhance the sensitivity of glioma to radiotherapy and chemotherapy by interfering with multiple signaling pathways, which also shows important application potential (Mo et al., 2022; Rahman et al., 2026). In addition to the above strategies, intervention against the immunosuppressive microenvironment of glioma has also become a research hotspot to overcome tumor resistance. By regulating tumor associated macrophages (TAMs) to reshape immune homeostasis, it provides a new adjuvant treatment idea for improving the effect of traditional treatment (Liu and Yu, 2025); The breakthrough in gene therapy provides a new technical support for the reversal of drug resistance. Gene delivery system based on non viral lipid carrier is becoming an intervention method with great transformation potential with the advantage of accurately correcting the genetic variation related to drug resistance (Luiz et al., 2021; Li H. et al., 2025).

In conclusion, glioma drug resistance is a complex network composed of efflux transporters, tumor stem cells, epigenetic and transcriptional regulation, abnormal signaling pathways and tumor microenvironment. It will provide new ideas and directions for optimizing the clinical diagnosis and treatment of glioma and improving the prognosis of patients.

## 2 Multidrug resistance transporter and its regulatory mechanism

### 2.1 P-glycoprotein in drug resistance of glioma

As a member of ATP binding cassette transporter family, P-glycoprotein plays a key role in the mechanism of glioma drug resistance. It can reduce the effective drug concentration in cells through active efflux of chemotherapy drugs, and ultimately lead to the decline of therapeutic effect. This transmembrane efflux pump can transport a wide range of substrates including a variety of anticancer drugs across the membrane by ATP hydrolysis. In glioma cells (especially in glioblastoma multiforme), P-glycoprotein is often highly expressed, which is also an important reason for the formation of clinical multidrug resistance phenotype. Enhanced efflux of P-glycoprotein will reduce the effective intracellular concentration of doxorubicin (DOX) and other drugs, resulting in reduced therapeutic response (Zhang Y. et al., 2023; Zhuo et al., 2024). The inherent drug resistance of glioma stem cells, as a key cell subset driving tumor occurrence, progression and recurrence, further weakens the therapeutic effect and significantly increases the difficulty of clinical treatment (Dogra et al., 2024).

The expression and activity of P-glycoprotein (P-gp) in glioma are affected by multiple factors of tumor microenvironment and associated signaling pathways. For example, PI3K/Akt signaling axis has been confirmed to be involved in the regulation of P-gp expression, and excessive activation of this pathway will increase the level of P-gp, thus promoting chemotherapy resistance. Resveratrol, a natural compound, has been shown to inhibit the PI3K/Akt pathway and regulate the expression of P-gp, which helps to enhance the sensitivity of glioma cells to chemotherapy drugs (Zhang Y. et al., 2023). This reveals the mechanistic relationship between the intracellular signal cascade and the regulation of drug efflux transporters. In addition, cytokines and growth factors secreted by tumor associated macrophages (TAMs) in the glioma microenvironment can indirectly regulate the expression of P-gp, thus forming a microenvironment conducive to drug resistance (Liu and Yu, 2025).

It is worth noting that the high expression of P-gp in blood-brain barrier and blood-brain tumor barrier (BTB) will significantly limit the effective penetration of chemotherapy drugs into the central nervous system and tumor tissues. The barrier effect of P-gp in the blood-brain barrier further aggravates the difficulty of glioma drug delivery. At present, new drug delivery systems that can bypass or inhibit the function of P-gp in the barrier are becoming a research hotspot. For example, the nanoparticles drug delivery system co loaded with curcumin and other P-gp inhibitors has shown enhanced drug accumulation of glioma cells and improved therapeutic effect in preclinical models (Zhuo et al., 2024). In addition, by engineering the bacterial mediated delivery platform to silence the expression of P-gp in tumor cells, it can not only enhance the sensitivity of glioma to chemotherapy, but also promote the penetration of blood-brain barrier (Mi et al., 2023).

At the same time, the expression of P-gp is also dynamically regulated by epigenetic and transcriptional levels, and this kind of regulation process is easily affected by some therapeutic interventions. For example, long-term temozolomide (TMZ) treatment can induce abnormal high expression of P-gp, and then mediate the occurrence of acquired drug resistance in glioma. However, studies have shown that mifepristone and other drugs combined with TMZ can reduce the level of P-gp, enhance the retention of drugs in tumor cells, and improve the survival outcome of glioma models (Llaguno-munive et al., 2020). In addition, naringenin and other natural compounds have been reported to improve the sensitivity of glioma cells to chemotherapy drugs (Jayalakshmi and Vanisree, 2020; Wang et al., 2023). These findings suggest that targeted regulation of P-gp expression and activity by regulating tumor microenvironment, signaling pathways and adjuvant drugs is expected to overcome the drug resistance of glioma.

In glioma stem cells, P-gp not only mediates chemotherapy resistance, but also plays an important role in maintaining stem cell characteristics and tumorigenic potential. The co expression of P-gp, multidrug resistance associated protein 1 (MRP1) and other drug resistance proteins, as well as the involvement of circular RNA (*circRNA*) regulating these proteins, further complicate the drug resistance regulatory network. For example, *circEPHB4* can regulate the expression of P-gp through the miRNA sponge mechanism, thereby affecting the sensitivity of glioma cells to temozolomide (TMZ) (Liao Y. et al., 2025). These evidences suggest that P-gp has been integrated into a multi-level molecular regulatory network to jointly participate in and determine the drug resistance phenotype of tumors.

In general, P-gp can actively expel chemotherapeutic drugs and interact with multiple signal transduction and regulatory pathways, which is the core molecule mediating glioma drug resistance. Its differential expression in tumor cells and stem cell subsets, as well as its wide distribution in the blood-brain barrier/blood-brain tumor barrier (BBB/BTB), jointly highlight the multiple challenges faced by targeting the transporter. The combined use of P-gp inhibition, upstream signaling pathway regulation and advanced drug delivery system is expected to overcome drug resistance through multiple mechanisms and improve the therapeutic effect of glioma. The development of such strategies should fully consider the high heterogeneity within glioma, so as to maximize the clinical benefits (Jovanović et al., 2020).

### 2.2 Multidrug resistance associated protein 1 and its signal regulation

Multidrug resistance associated protein 1 (MRP1) is encoded by ABCC1 gene and belongs to ATP binding cassette (ABC) transporter family. It is highly expressed in glioblastoma multiforme cells. As an efflux pump, MRP1 can actively transport a variety of chemotherapy drugs from cancer cells, thereby reducing the accumulation of drugs in cells and promoting multidrug resistance (Padder, 2025). Its overexpression in GBM is the key mechanism leading to temozolomide and other standard chemotherapeutic drugs resistance, which will not only limit the clinical therapeutic effect, but also significantly worsen the prognosis of patients. The ability to transport chemotherapeutic drugs *in vitro* enables tumor cells to obtain the advantages of reducing cytotoxicity and survival; It is worth noting that drug transport mediated by MRP1 usually depends on glutathione (GSH), and regulating GSH binding can affect MRP1 activity and drug resistance (Hanssen et al., 2022). Recent studies have also found that small molecule inhibitors and regulators targeting MRP1 can effectively overcome MDR of cancer cells, including glioma, which also highlights the clinical value and application prospect of MRP1 as a potential therapeutic target for drug resistance intervention (Čičak et al., 2026).

In addition, the exonuclease CD73 and its product adenosine (ADO) have been confirmed to be involved in regulating the expression and function of MRP1. CD73 can regulate the level of MRP1 in cancer cells through adenosine A3 receptor (A3R) - mediated adenosine signal transduction, thereby affecting drug resistance. For example, in cervical cancer cells, ADO positively regulates MRP1 expression in a dose-dependent manner, while inhibiting CD73 or blocking A2A receptor will reduce MRP1 expression and enhance cisplatin sensitivity (Carrera-Martínez et al., 2023). This signal axis indicates that purinergic signal transduction is involved in regulating the expression of MRP1 and MDR phenotypes in tumors. A similar mechanism may also exist in GBM, whose tumor microenvironment is rich in extracellular adenosine, which may regulate the expression of MRP1 by activating adenosine receptor signaling pathway, and then regulate the drug resistance of glioma cells.

Studies have confirmed that PI3K/Akt and MEK/ERK signaling pathways play a key role in the expression and functional regulation of MRP1. In osteosarcoma and leukemia models, activation of PI3K/Akt pathway can upregulate the expression of MRP1, thereby enhancing drug efflux and mediating tumor resistance; On the contrary, inhibition of this pathway can reduce the level of MRP1 and improve the sensitivity of cancer cells to chemotherapy. For example, naringenin, a natural flavonoid, downregulates MRP1 by inhibiting PI3K/Akt pathway, thereby enhancing the sensitivity of drug-resistant osteosarcoma cells to cisplatin; Similarly, by inhibiting the PI3K/Akt/MRP1 axis, the silencing of CRKL can reverse the resistance of leukemia cells to doxorubicin (Zhao et al., 2021; Yu et al., 2021). MEK/ERK pathway is also involved in the regulation of MRP1, which can affect the stability and function of MRP1 through transcriptional regulation and post-translational modification; These signal cascades can integrate extracellular signals and intracellular stress, and then accurately regulate the drug resistance process mediated by MRP1.

In glioma, the transcriptional regulation of MRP1 involves a variety of key molecules, including the transcription factor *RUNX1*, which can be negatively regulated by mir-128-3p. Studies have confirmed that *RUNX1* upregulation can promote the expression of MRP1 in GBM cells and enhance TMZ resistance, while mir-128-3p can enhance the chemotherapy sensitivity of tumor cells to TMZ by targeted inhibition of *RUNX1* (Xu et al., 2021b). In addition, circular RNA (such as *circVPS18*) can release its inhibitory effect on *RUNX1* by adsorbing mir-370, and then participate in the regulation of TMZ resistance (Li W. et al., 2021). The regulation of MRP1 also involves RNA apparent modification. For example, *METTL3* mediated m6A methylation can enhance the translation efficiency of MRP1 mRNA and promote imatinib resistance in gastrointestinal stromal tumors (Xu et al., 2022). These studies reveal that the formation of chemotherapy resistance in glioma depends on a complex network composed of post transcriptional regulation and epigenetic modification, and regulates the expression and function of MRP1 from multiple dimensions.

At present, the structure and biochemical characteristics of MRP1 have been clearly clarified, which lays a foundation for the development of high-efficiency inhibitors. MRP1 is composed of multiple transmembrane domains and nucleotide binding domains, and its key amino acid residues play a decisive role in protein expression and function (Conseil and Cole, 2021; Smith et al., 2021). Molecular docking and computer simulation studies show that dietary flavonoids can bind to the nucleotide binding domain 1 (NBD1) of MRP1, thereby inhibiting its drug efflux activity (Padder, 2025). New inhibitors such as tetrahydroquinoline/4,5-dihydroisoxazoline hybrid showed strong inhibitory effect in MRP1 overexpression cell model and could effectively reverse drug resistance (Da Silva Pereira et al., 2025). In addition, macrocyclic peptide compounds can maintain MRP1 in a catalytically inactive conformation, providing a new strategy for related treatment (Pietz et al., 2023). These research advances indicate that targeted regulation of MRP1 is expected to overcome the multidrug resistance (MDR) of GBM and other tumors.

In conclusion, MRP1 is highly expressed in GBM and a variety of tumors, which can promote tumor multidrug resistance (MDR) by mediating the efflux of chemotherapeutic drugs. CD73 adenosine signaling regulates MRP1 expression through A3 receptors, and PI3K/Akt and MEK/ERK pathways are key downstream regulators of MRP1 activity. Transcription factors such as *RUNX1* and non coding RNA affect the expression of MRP1, and epigenetic modification further fine regulates its function. The analysis of MRP1 structure is helpful to design specific inhibitors to resist drug resistance. These studies systematically elucidated the complex regulatory network of MRP1 in glioma chemotherapy resistance, and provided important potential targets for improving the clinical therapeutic effect. More clinical translational studies are still needed in the future to develop efficient MRP1 targeted therapy strategies for patients with GBM.

### 2.3 Nuclear localization and function of breast cancer resistance protein in drug resistance

Breast cancer resistance protein (ABCG2) is an important ATP binding cassette transporter. Its role in mediating multidrug resistance in breast cancer and a variety of malignant tumors has been widely confirmed. The traditional view is that BCRP, as a plasma membrane efflux transporter, can actively pump chemotherapeutic drugs out of the cell, reduce intracellular drug accumulation, and then reduce the therapeutic effect (Zattoni et al., 2022). However, the latest research evidence shows that BCRP is not only located in the plasma membrane, but also can be distributed in the nucleus, suggesting that in addition to the classic drug efflux function, BCRP may also be involved in other biological processes.

The nuclear localization of BCRP has been confirmed in a variety of tumor cells such as breast cancer, and it can participate in the process of tumor drug resistance at the level of genome regulation. This unique subcellular distribution suggests that BCRP may play a role by protecting the nucleus from cytotoxic substances or regulating the gene expression of drug resistance related signaling pathways. For example, the study of immunofluorescence and subcellular fractionation technology has confirmed that BCRP can exist in nuclear components (Lye et al., 2022). Such nuclear BCRP may promote the efflux of chemotherapeutic drugs from the nucleus, reduce DNA damage, and then participate in the formation of tumor resistance.

Studies have shown that inhibition of BCRP function can significantly enhance drug sensitivity and improve the effect of chemotherapy. Drug inhibitors such as ilakeda have been proved to effectively block the activity of BCRP, thereby increasing the intracellular accumulation of topotecan and other substrates in two-dimensional and three-dimensional ovarian cancer cell models (Stasiak et al., 2025). These inhibitors can restore drug efficacy by overcoming the drug efflux effect of BCRP, including its drug isolation function in the nucleus, which may be the key mechanism to reverse drug resistance. In addition, new carborane targeted BCRP inhibitors have shown good effects in enhancing the sensitivity of cancer cells to chemotherapy drugs such as adriamycin and cisplatin (Paskas et al., 2023).

Regulating the expression and localization of BCRP involves complex transcriptional and post transcriptional regulatory networks. Nuclear receptors (such as CXR) can upregulate the expression of BCRP, and its agonists can enhance the expression level and transport activity of BCRP in hepatocytes (Xu et al., 2023); Quercetin and other natural compounds can inhibit CXR mediated BCRP transcriptional activation, reduce its expression and increase drug uptake, which has been confirmed in chicken models (Deng et al., 2025). These studies suggest that nuclear receptor signaling pathway can regulate the expression, localization and function of BCRP, and then participate in the formation of tumor drug resistance phenotype.

In addition, the interaction between BCRP and NF - κ B, p53 and other key signaling pathways has been confirmed to be involved in the regulation of drug resistance. For example, β - sitosterol can inhibit the expression of BCRP through the p53/nf- κ B signal axis, thereby reversing multidrug resistance (Wang Z. et al., 2020); The drug resistance of breast cancer cells to zoledronic acid and other drugs is related to the high expression of BCRP and the activation of NF - κ B pathway, and the increase of NF - κ B level in the nucleus is significantly positively correlated with the increase of BCRP (Dikkatli et al., 2025).

In conclusion, the nuclear localization of BCRP is an important feature of BCRP mediated tumor resistance. This special subcellular distribution expands the role of BCRP in chemotherapy resistance, which not only participates in drug efflux at the plasma membrane level, but also may play a role in nuclear protection and transcriptional regulation. Targeting intranuclear BCRP and synergistic intervention with membrane-bound form are expected to provide a more comprehensive strategy for reversing multidrug resistance and improving the effect of chemotherapy. In order to further tap its clinical therapeutic potential, it is still necessary to further analyze the molecular mechanism regulating the expression and function of BCRP (Mehendale-Munj and Sawant, 2021).

### 2.4 Regulation mechanism of multidrug resistance transporter expression

The expression of multidrug resistance transporters (such as P-glycoprotein/P-gp/ABCB1, multidrug resistance associated proteins and breast cancer resistance proteins) is strictly regulated by gene transcription, epigenetic modification and post transcriptional regulation. Transcription factors play a key role in the regulation of the expression of these transporters. For example, zinc finger protein family members such as C2H2 transcription factor zfpa in Aspergillus fumigatus can negatively regulate the expression of drug efflux pump genes (such as atrf), thereby affecting the sensitivity of azole drugs (Li Y. et al., 2023). Similarly, the transcription factor ETV1 can activate *METTL3* transcription, mediate N6 methyladenosine (m6A) modification of MRP1 mRNA, enhance its translation efficiency, and ultimately promote imatinib resistance in gastrointestinal stromal tumors (Xu et al., 2022). These studies highlight the important role of transcriptional regulators in the dynamic regulation of MDR transporter gene expression.

Epigenetic modifications (especially histone acetylation) play an important role in regulating the expression of MDR transporters. Histone deacetylase inhibitor (HDACi) can improve chromatin accessibility by enhancing histone acetylation level, and then regulate the expression and function of MDR1 and BCRP (You et al., 2020). This epigenetic regulation exists not only in tumor cells, but also in non malignant tissues, suggesting that histone modification has a wide range of physiological significance in the regulation of transporters. In addition, microRNAs (miRNAs) have become key molecules for post transcriptional regulation of MDR transporters. For example, the reduced expression of mir-873-5p under hypoxic conditions can lead to the upregulation of MDR1 and pregnane X receptor (PXR), and then affect the function of transporters and drug metabolism (Duan et al., 2022). MiRNA mediated regulation further enriches the complexity of drug resistance regulatory network, and integrates hypoxia and other microenvironment signals into the expression regulation of transporters.

Tumor microenvironment, especially hypoxia and inflammation, has a profound impact on the expression of MDR transporters. Hypoxia inducible factors (HIFs) and inflammatory cytokines can regulate the transcription of transporter genes through direct action or through intermediate regulatory factors such as nuclear receptors. In addition, the characteristics of tumor stem cells can also drive the abnormal high expression of transporters. For example, in glioblastoma stem cells, the multidrug and toxin efflux transporter *SLC47A1* is highly expressed and is associated with poor prognosis, and its expression level is associated with the pathways supporting tumor growth and survival (Batara et al., 2023). Inflammatory states such as pregnancy complications can also change the expression of placental efflux transporters, which may damage the barrier function and affect fetal protection (Kozlosky et al., 2022). These observations indicate that many factors, such as microenvironment stress and characteristics of tumor stem cells, can induce adaptive changes in the expression of transporters, leading to drug resistance.

**FIGURE 1:**
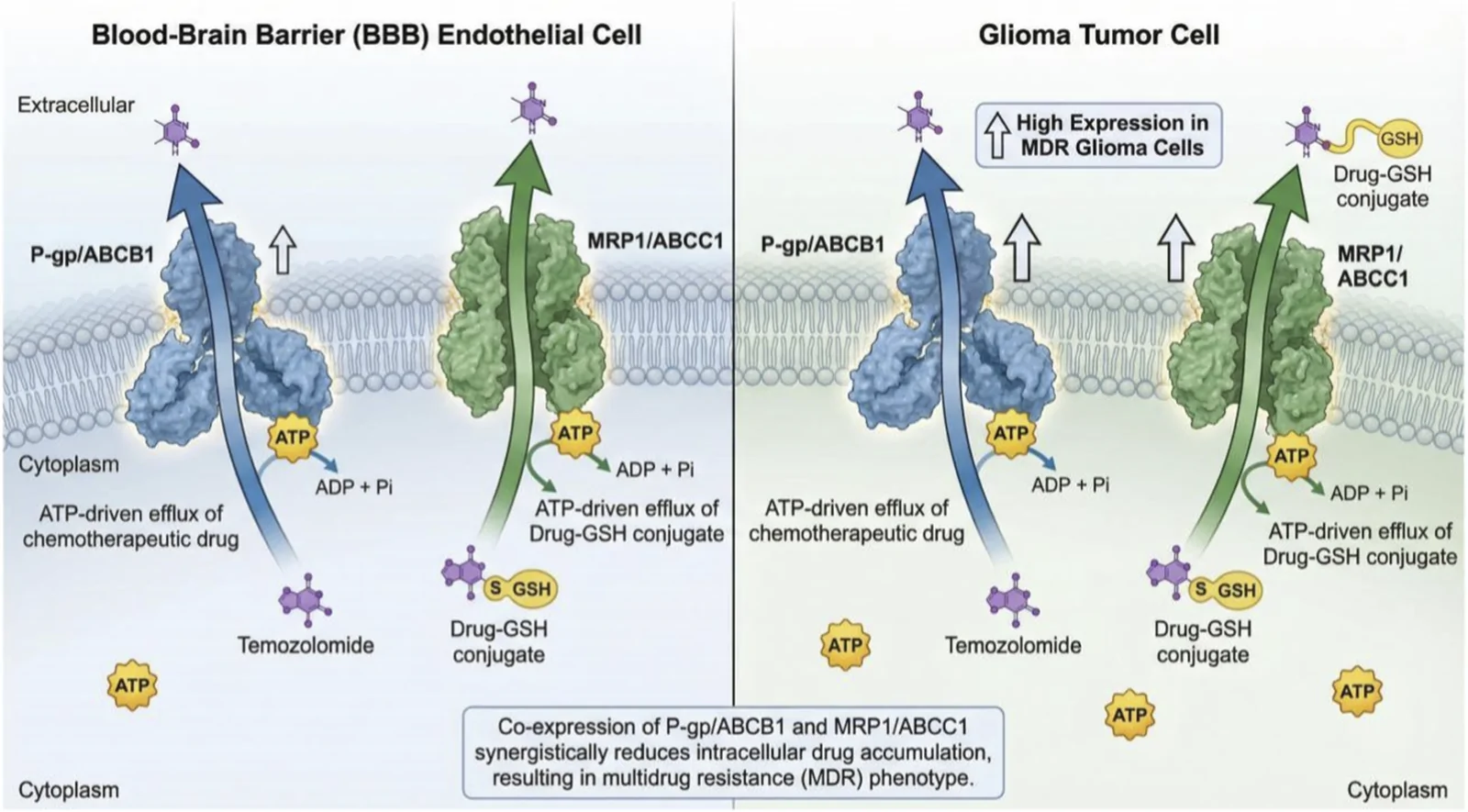
Drug efflux pump (MRP1, P-gp), DNA repair protein (*MGMT*) and hippo/Notch signaling pathway jointly regulate the intracellular drug level, DNA damage repair and stem cell stemness, and promote tumor resistance and survival.

Scaffolds and transcription factors can synergistically regulate the function and expression of MDR transporters. Snai transcription factor family, composed of snail, slug and SMUC, can regulate the subcellular localization and activity of transporters by interacting with ERM proteins (ezrin, Radixin, moesin), and then participate in the expression regulation of P-glycoprotein (P-gp), multidrug resistance protein 2 (MRP2) and BCRP. For example, in lung cancer cells, snail can regulate the activity of P-gp by combining moesin, revealing a new mechanism of regulating drug efflux through the synergistic effect of transcriptional regulation and scaffold protein (Zhang et al., 2021). This synergistic effect of transcription factors and scaffold proteins may extend to other MDR transporters and different cancer types, suggesting that there is a conservative regulation axis integrating transcription factors and cytoskeletal components, which can control the function of MDR transporters through multi-level effects and participate in the regulation of tumor drug resistance (Figure 1).

These regulatory mechanisms together constitute a complex regulatory network, which precisely regulates the expression and activity of MDR transporters through multiple pathways such as transcription factors, epigenetic modifications, non coding RNA and tumor microenvironment factors. This multi-level precise regulation not only gives tumor cells the ability to adapt to the pressure of chemotherapy, but also provides rich and key therapeutic targets for reversing multidrug resistance. For example, targeting transcription activators such as ETV1 or intervening with epigenetic regulatory molecules such as histone deacetylases (HDACs) can effectively regulate the expression of transporters and significantly enhance the sensitivity of tumor cells to chemotherapy drugs. In addition, in-depth elucidation of the regulatory role of tumor microenvironment on transporters will provide new ideas for the development of joint treatment strategies, and further improve the clinical therapeutic effect by blocking such adaptive drug resistance pathways.

## 3 Tumor stem cells and glioma drug resistance

### 3.1 Drug resistance characteristics of glioma stem cells

Glioma stem cells have unique biological characteristics such as self-renewal and multi-directional differentiation, which are the core basis of glioma occurrence, progression and recurrence. Its strong stem maintenance and cell plasticity have become the main challenges in the clinical treatment of glioma (Król et al., 2020). The self-renewal ability of glioma stem cells enables them to refill the tumor body after active treatment, which has become a key target for reversing tumor drug resistance. Studies have confirmed that glioma stem cells highly express Sox2, Oct4, *NANOG* and other stem related transcription factors, which play an important role in maintaining the undifferentiated state of cells and mediating resistance to conventional treatment (Wang et al., 2021b). This stem cell like phenotype is closely related to enhanced DNA repair ability and activation of survival signaling pathway, which further aggravates the complexity of glioma treatment.

One of the key mechanisms of drug resistance in glioblastoma stem cells (GSCs) is the abnormal high expression of multidrug resistance transporters (especially ATP binding cassette transporters, such as *ABCB4* and ABCA1). These efflux transporters can actively pump chemotherapeutic drugs such as temozolomide (TMZ) out of the cell, reduce the intracellular drug concentration and weaken the therapeutic effect. For example, *ABCB4* is highly expressed in GSCs and can be transmitted to differentiated glioma cells through exosomes, thus promoting the spread of chemotherapy resistance in the tumor microenvironment (Xu et al., 2024). Transcription factor ATF3 has been confirmed to regulate the expression of *ABCB4*, revealing a transcriptional regulation axis that mediates the maintenance of drug resistance phenotype. Similarly, ABCA1 was upregulated in GSCs and involved in TMZ efflux; Inhibition of ABCA1 can significantly enhance the apoptosis of GSCs induced by TMZ, suggesting that targeting the above transporters is expected to reverse the chemotherapy resistance of glioma cells (Wang et al., 2021b). These findings highlight the complexity of GSCs' resistance mechanism, and confirm that ABC transporter is a potential intervention target in glioma treatment.

In addition, glioblastoma stem cells (GSCs) also exhibit unique metabolic adaptability to support their survival under chemotherapy stress. Metabolic profile analysis showed that GSCs were highly dependent on pyruvate carboxylase (PC) and other specific enzymes to maintain their viability and self-renewal characteristics. Inhibition of PC can not only directly induce the apoptosis of GSCs, but also restore their sensitivity to etoposide and other chemotherapeutic drugs, which suggests that the metabolic vulnerability of GSCs can be targeted and utilized, becoming an important breakthrough to overcome tumor resistance (Renoult et al., 2024). At the same time, compared with differentiated glioma cells, GSCs had obvious mitochondrial dysfunction, the content and activity of mitochondria were significantly reduced, but the sensitivity to mitochondrial inhibitors was significantly increased. Studies have shown that inhibitors such as oligomycin a, antimycin A and rotenone can selectively induce apoptosis or autophagy of GSCs (Datta et al., 2021). These unique metabolic characteristics provide a solid theoretical basis for the development of new therapeutic strategies that target the destruction of GSCs' specific energy generation pathway and reverse their drug resistance.

The abnormal activation of tumor microenvironment and related signaling pathways also significantly regulated the drug resistance phenotype of glioblastoma stem cells (GSCs). Taking Notch signaling pathway as an example, this pathway plays a key regulatory role in maintaining the dry characteristics of GSCs and mediating their drug resistance. Downregulation of Notch signaling pathway can significantly enhance the sensitivity of GSCs to chemotherapy drugs by regulating the expression of drug transporters and affecting the process of epithelial mesenchymal transition (EMT) (D’amico and De Amicis, 2022). In addition, integrins highly expressed on the surface of GSCs can promote the self-renewal ability of cells by mediating cell adhesion and downstream signal transduction, and enhance their resistance to radiotherapy and chemotherapy, which suggests that the treatment strategy targeting integrins is expected to destroy the above protective interactions and reverse drug resistance (Wang M. et al., 2022). It is worth noting that GSCs have significant heterogeneity, including subtypes with different stemness scores and molecular characteristics. This heterogeneity further increases the difficulty of clinical treatment, because it leads to different survival outcomes and drug resistance characteristics in different GSC populations (Abdelrahman et al., 2024).

In conclusion, glioblastoma stem cells (GSCs) mediate glioma drug resistance through multi-level and networked regulatory mechanisms, including enhanced self-renewal ability, abnormal upregulation of drug efflux transporters, adaptive changes of metabolic phenotypes, and transmission of supportive signals in the tumor microenvironment. These core characteristics not only significantly reduce the sensitivity of GSCs to conventional chemotherapy, but also become the key driver of tumor recurrence. Targeting intervention against the unique biological characteristics of GSCs, such as ABC transporter mediated drug efflux, metabolic vulnerability, and key signaling pathways such as notch and integrin, is expected to improve the therapeutic effect of glioma. In addition, in view of the significant heterogeneity of GSCs population, personalized treatment strategies may be needed in the future to fully consider the specific drug resistance mechanism of different GSC subtypes, so as to effectively improve the treatment outcome and optimize the clinical prognosis of glioma patients (Xu et al., 2024; Renoult et al., 2024).

### 3.2 Role of cell signaling pathway in drug resistance of glioma stem cells

Glioma stem cells are a subset of cells with self-renewal and multi-directional differentiation potential in glioma tissue, and play a central role in the formation of tumor heterogeneity, malignant progression and chemoradiotherapy resistance. The mechanism of drug resistance mediated by these stem cells is extremely complex, involving many signaling pathways that regulate stem cell characteristics, cell viability and stress response. Among them, adenosine receptor signaling pathway and Hippo pathway have been proved to be the key molecular pathways to regulate the drug resistance of glioma stem cells, and also an important breakthrough in the current research of glioma targeted therapy.

Adenosine signaling, especially adenosine A3 receptor (A3AR) - mediated signaling pathway, plays a key role in enhancing the chemoresistance of glioblastoma stem cells (GSCs). In the hypoxic tumor microenvironment, the increase of extracellular adenosine content will activate A3AR, and then upregulate the expression of MRP3 and other multidrug resistance related proteins, enhance the efficiency of chemotherapy drug efflux, reduce intracellular drug accumulation, and ensure the survival of GSCs during treatment. Previous studies have confirmed that hypoxia can also induce the expression of MRP3 through A2B adenosine receptor and participate in the formation of drug resistance of glioma cells (Rocha et al., 2022). After activation of A3AR signal axis, it will further regulate the expression of downstream transcription factors and drug transporter genes, and various changes will synergize, strengthen the drug efflux function of GSCs, and finally form a stable chemotherapy resistance phenotype. Targeted intervention against this pathway has also become a potential direction of reverse transformation therapy resistance.

Similar to the adenosine receptor signaling pathway, the abnormal activation of Hippo pathway is also closely related to the stem maintenance and treatment resistance of glioblastoma stem cells (GSCs). Hippo pathway was originally a classic pathway to regulate organ size and cell proliferation. Once it was out of balance, its downstream effector molecules *YAP* and TAZ would aggregate in the nucleus and start the transcription process to promote the maintenance of stem cell characteristics, cell survival and proliferation. In glioma, the abnormality of Hippo pathway is not only directly related to tumor malignant progression and treatment resistance (Wu H. et al., 2022), but also interacts with PI3K/Akt, wnt/β-catenin and other carcinogenic pathways, further stabilizing the drug resistance phenotype of GSCs. The activation of this pathway can upregulate the expression of drug transport genes and anti apoptotic factors, induce multidrug resistance, and inhibit the activity of key components of Hippo pathway or Yap/TAZ, which can effectively reduce the activity of GSCs and improve their sensitivity to treatment, highlighting the therapeutic value of this pathway.

The drug resistance phenotype of glioma stem cells (GSCs) is not the result of a single pathway regulation, but is determined by a multi-level pathway network that maintains the characteristics of stem cells, regulates drug efflux, and transmits survival signals. Adenosine A3 receptor mediated upregulation of MRP1 and other efflux transporters, which realized the active discharge of chemotherapy drugs; The disorder of Hippo pathway has maintained the stem cell characteristics and drug resistance characteristics of GSCs for a long time, and the two pathways can further strengthen the drug resistance effect by cross-linking and converging on common downstream targets. At the same time, the dynamic tumor microenvironment, such as hypoxia and inflammation, can regulate the above pathways. For example, hypoxia induces adenosine accumulation to activate A3AR signal, and mechanical signals and intercellular interactions regulate the activity of Hippo pathway. This microenvironment dependent regulation further aggravates the drug resistance of GSCs. In conclusion, adenosine A3 receptor signaling and abnormal activation of Hippo pathway are the core links of regulating GSCs' drug resistance. The combination of the two pathways is expected to produce synergistic therapeutic effect and overcome stem cell-mediated drug resistance. In the future, we need to further explore the cross regulation mechanism between pathways, clarify its internal relationship with other drug resistance pathways, and lay the foundation for the development of accurate and effective glioma treatment (Rocha et al., 2022).

### 3.3 Interaction between GSCs and tumor microenvironment

Glioma stem cells (GSCs) are mainly located in a special microenvironment characterized by hypoxia. This microenvironment not only determines its biological characteristics, but also is the key structural basis for mediating therapeutic resistance. Hypoxia can significantly upregulate the expression of genes related to stem cell maintenance, cell survival and drug resistance in GSCs. Hypoxia inducible factors (HIFs) are stably activated under hypoxic conditions, which can continuously maintain the stem phenotype of GSCs and enhance treatment resistance (Boyd et al., 2021). In addition to directly regulating gene transcription, hypoxia can also reshape the extracellular matrix (ECM) and induce stromal cells such as astrocytes to secrete tumor promoting factors, further supporting the dry maintenance and invasive ability of GSCs (Nicholson et al., 2024). A positive feedback loop is formed between GSCs and the hypoxic microenvironment: hypoxia directly regulates the function of GSCs, and indirectly promotes tumor progression and drug resistance through remodeling the microenvironment, so that it can still survive under high-intensity treatment, which has become an important source of tumor recurrence.

In addition to hypoxia adaptive changes, GSCs can also actively interact with immune cells, endothelial cells, mesenchymal stem cells (MSCs) and other components in the tumor microenvironment to construct a microenvironment with immunosuppressive and tumor promoting properties. GSCs can upregulate the secretion of histamine through h3k4me3 dependent histidine decarboxylase (HDC) driven by Myc, act on endothelial cells, promote angiogenesis through the histamine H1 receptor Ca ^2^+NF κ b axis, and accelerate tumor angiogenesis and malignant progression (Chen et al., 2022); At the same time, GSCs release extracellular vesicles rich in cluster proteins, which can induce tumor associated macrophages to polarize to M2 type and enhance the chemoresistance to temozolomide (Wen et al., 2025). These interactions not only maintain the dryness of GSCs stably, but also reshape the immune microenvironment to mediate immune escape, which further aggravates tumor resistance and malignant progression.

**FIGURE 2:**
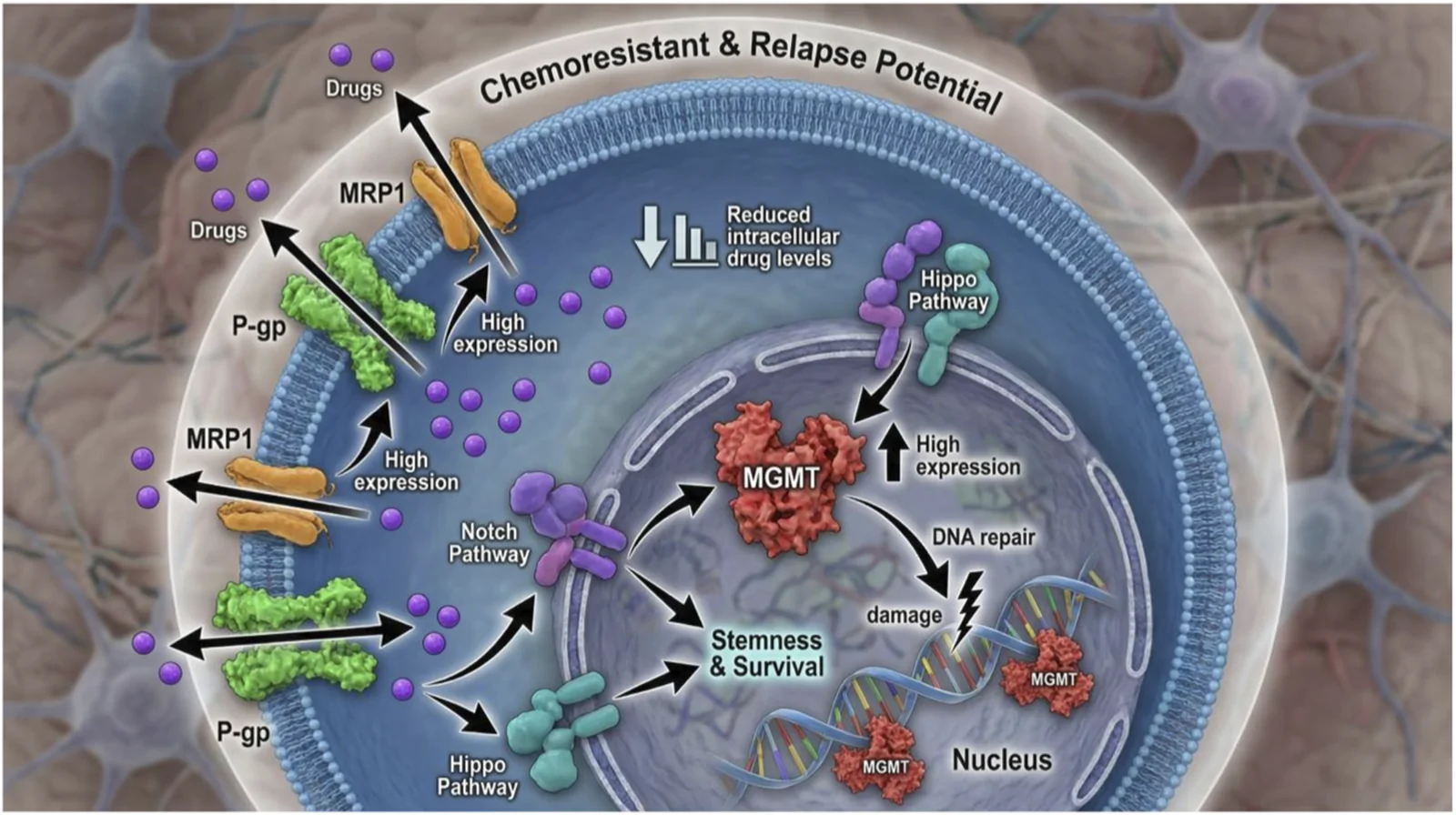
Molecular mechanisms of glioma cell chemoresistance and recurrence: transport proteins, Notch/Hippo pathway, and MGMT-mediated DNA repair jointly drive the chemoresistant stem-like phenotype.

The interaction between GSCs and tumor associated macrophages (TAMs) has a more refined molecular regulation mechanism. GSCs can induce TAMs to highly express glycoprotein NMB (GPNMB), which promotes glycolytic metabolism and self-renewal of GSCs by activating pyk2/rsk2 pathway (Liu et al., 2025), and blocking GSC TAM interaction is expected to become a potential therapeutic strategy. Similarly, the *USF1/CD90* axis can enhance the physical adhesion between GSCs and macrophages, promote the polarization of TAMs to immunosuppressive phenotypes, and maintain the dryness of GSCs to promote tumor progression (Zhou Y. et al., 2022). These findings suggest that the complex regulation of GSCs on immune cells in the microenvironment is an important part of tumor immune escape and treatment resistance. In addition, the plasticity of GSCs enables them to dynamically respond to microenvironment signals such as hypoxia and immunity. Abnormal expression of epigenetic regulatory factors (methylase, demethylase, histone deacetylase) can lead to transcriptional reprogramming disorders, and regulate the phenotypic transition of GSCs between neural precursor like and mesenchymal like States, which is closely related to the increase of tumor malignancy and treatment resistance (Uribe et al., 2022). Hypoxia inducible factor can also affect the polarization of TAMs by regulating the secretion of TREM1 and TGF β 2, promote the transformation of GSCs to mesenchymal like phenotype and strengthen the immunosuppressive microenvironment (Dong et al., 2024), suggesting that hypoxia signal and immune regulation form a highly intertwined regulatory network, and jointly maintain the malignant phenotype of GSCs (Figure 2).

In conclusion, targeting the hypoxic microenvironment and blocking the interaction between GSCs and immune cells is expected to improve the therapeutic effect of glioma. Drug intervention of histamine receptor and inhibition of extracellular vesicle communication can block tumor promoting signals, and targeting epigenetic factors related to GSCs plasticity can also enhance treatment sensitivity. However, GSCs subgroups are highly heterogeneous, and their interactions with the microenvironment are complex and diverse. In the future, it is necessary to develop individualized treatment strategies that take into account the spatio-temporal dynamics of the tumor ecosystem. Based on the existing evidence, GSCs can not only survive and proliferate in the hypoxic microenvironment, but also actively reshape the microenvironment to promote immune escape, angiogenesis and drug resistance. In depth analysis of these multi-level interaction mechanisms can provide a theoretical basis for the development of new combined treatment strategies. Its core goal is to destroy the protective microenvironment of GSCs, so as to effectively overcome glioma resistance.

## 4 Effect of tumor microenvironment and hypoxia on drug resistance of glioma

### 4.1 Formation of hypoxic microenvironment and its biological effects

Gliomas, especially high-grade gliomas such as glioblastoma multiforme, grow much faster than the development and neogenesis of the vascular system, thus forming local hypoxic areas in the tumor microenvironment. Hypoxia is the key link to drive malignant progression of glioma and induce treatment resistance. Local hypoxic environment can promote the stable activation of hypoxia inducible factors. HIF-1 α and HIF-2 α, as core transcription regulators, mediate the adaptive changes of tumor cells to hypoxic microenvironment. For example, after HIF-1 α activation, it can upregulate the transcription expression of stem cell characteristics and chemotherapy resistance related genes, and further enhance the invasion ability of glioma (Wang P. et al., 2022).

Hypoxia can induce the metabolic reprogramming of glioma cells, making their energy production shift from oxidative phosphorylation to glycolysis. This phenomenon is called the Warburg effect. This metabolic transition is mainly regulated by hypoxia inducible factor-1 α (HIF-1 α), which can upregulate glycolytic enzymes and glucose transporters (such as GLUT1 and *LDHA*), thus enhancing glucose uptake and lactate production under aerobic conditions. Under hypoxic conditions, the upregulation of the key glycolytic enzyme pyruvate kinase M2 (*PKM2*) supports this metabolic adaptation (Li G. et al., 2024). In addition, studies have found that RLIP76 is a hypoxia inducible molecule, which can stabilize HIF-1 α and promote glycolysis and tumorigenesis of glioma cells (Wang Q. et al., 2022).

Hypoxia microenvironment can also directly regulate the biological behavior of glioma stem cells. As a key cell subgroup closely related to tumor occurrence, progression and recurrence, the stemness and drug resistance of glioma stem cells (GSCs) are deeply regulated by hypoxia microenvironment. Hypoxia can induce glioma cells to dedifferentiate into GSCs through HIF1 α/hif2 α - Sox2 axis, and enhance their dry phenotype and chemotherapy resistance (Wang P. et al., 2022); In addition, factors such as TGFBI induced by hypoxia can stabilize EphA2 protein to maintain the characteristics of GSCs, further confirming the important role of hypoxia in maintaining tumor stemness potential (Chen et al., 2024).

Hypoxia not only directly affects tumor cells, but also can comprehensively reshape the tumor microenvironment by regulating the phenotypes of stromal cells and immune cells. Under hypoxic conditions, tumor associated astrocytes are abnormally activated, secreting a large number of tumor promoting cytokines such as TGF - β 1, IL-3, VEGF-A, and accelerating the malignant progression of glioma (Pantazopoulou et al., 2021); At the same time, hypoxia can drive tumor cells to release exosomes carrying miRNAs such as mir-25-3p and mir-1246. On the one hand, it can induce tumor associated macrophages to polarization to immunosuppressive M2 phenotype, activate PI3K Akt mTOR pathway to promote tumor development, on the other hand, it can also enhance the migration and invasion ability of normoxic glioma cells, and realize malignant regulation of adjacent cells (Xue et al., 2024; Qian et al., 2021). In addition, hypoxia drives the accumulation of lactic acid and other metabolic by-products in the tumor microenvironment (TME). After being ingested by macrophages, it can further induce M2 polarization by histone modification and upregulation of TNFSF9 expression. This metabolic epigenetic cross regulation clearly reveals how hypoxia mediated metabolic disorders shape the immunosuppressive microenvironment and promote glioma progression (Li M. et al., 2024).

At the molecular level, hypoxia can regulate the expression of a variety of non coding RNAs, including microRNAs and long non coding RNAs, which can widely affect the biological behavior of glioma cells and immune response. For example, hypoxia induced long non coding RNA *PDIA3P1* can bind mir-124-3p and activate NF - κ B pathway, thereby promoting the interstitial transformation of glioma cells and enhancing tumor invasion (Wang S. et al., 2020); Similarly, hypoxia related long non coding RNA can also construct tumor prognosis characteristics closely related to immunosuppression and treatment resistance (Li X. et al., 2024). Moreover, the hypoxic microenvironment can also induce glioma cells to undergo epithelial mesenchymal transition like processes through HIF-1 α, Gli1 and other transcription factor mediated signaling pathways, significantly enhancing cell migration and invasion (Lin and Guo, 2022); In addition, biological rhythm can also interfere with hypoxia related carcinogenic pathways, metabolic reprogramming and angiogenesis, and further affect the malignant progression of glioma (Le et al., 2024).

Hypoxia induced exosome loading also plays a key regulatory role, including a variety of bioactive substances such as circular RNA and microRNA, which can maintain tumor growth and mediate therapeutic resistance by regulating the function of receptor cells. For example, the upregulated exosome circ101491 under hypoxic environment can accelerate the malignant progression of glioma by regulating mir-125b-5p/edn1 axis (Zhang X. H. et al., 2023). This remote regulation mediated by extracellular vesicles further magnifies the tumor promoting effect of hypoxic microenvironment.

In conclusion, the formation of hypoxic microenvironment in glioma will trigger a series of complex biological effect networks, including transcriptional reprogramming, metabolic changes, stem maintenance, immune regulation and enhanced invasive potential. These changes are intertwined, which jointly promote tumor progression and induce treatment resistance, and ultimately lead to adverse clinical outcomes. An in-depth understanding of these regulatory mechanisms can provide a key basis for the development of targeted therapy, and then effectively block the malignant pathway driven by hypoxia in glioma (Wang P. et al., 2022; Chen et al., 2024). It is worth noting that the close interaction between hypoxia induced metabolic reprogramming and immune cell polarization suggests that targeted metabolic pathways or exosome communication may regulate the immunosuppressive microenvironment and improve the therapeutic response; However, considering the high heterogeneity of glioma subtypes and microenvironment conditions, more in-depth studies are still needed to clarify the precise molecular mediators in different glioma backgrounds and verify the feasibility of various potential therapeutic targets.

### 4.2 Role of adenosine signaling pathway in hypoxia induced drug resistance

Adenosine signaling pathway plays a key role in the occurrence and development of glioma drug resistance. Especially under the unique hypoxia condition of tumor microenvironment, the key enzyme CD73 in the pathway can catalyze the conversion of AMP to adenosine. The increase of CD73 activity will lead to the increase of extracellular adenosine concentration, and then activate adenosine receptors, especially the A3 receptor subtype. Studies have confirmed that A3 receptor activation can significantly enhance the function of multidrug resistance transporters such as ATP binding cassette (ABC) family, promote the active efflux of chemotherapy drugs, reduce the effective concentration of drugs in cells, and ultimately weaken the effect of chemotherapy. This mechanism is an important reason for the formation of multidrug resistance phenotype of glioma, and is also a key factor limiting the efficacy of traditional chemotherapy (Rocha et al., 2022). In hypoxic environment, glioblastoma stem cells can also upregulate drug resistance related transporters through adenosine signaling, for example, by activating A2B adenosine receptor to induce MRP3 expression, thereby enhancing drug efflux and promoting chemotherapy resistance; Knockdown of MRP3 or inhibition of A2B receptor signal can reduce the survival rate of glioma stem cells in hypoxic microenvironment and restore their sensitivity to chemotherapy drugs, suggesting that hypoxia, adenosine receptor activation and drug transporters form a regulatory axis to promote survival and drug resistance (Rocha et al., 2022).

In addition to directly regulating drug resistance of tumor cells, adenosine signaling also plays an important immunosuppressive function in the glioma microenvironment. The high concentration of extracellular adenosine mediated by CD73 can inhibit the anti-tumor immune response by acting on the adenosine receptor on the surface of immune cells, which shows that the activity of cytotoxic T cells decreases and the function of regulatory T cells increases, thus forming an immunosuppressive microenvironment, promoting tumor progression and reducing the therapeutic effect of immune checkpoint inhibitors. In glioblastoma, intervention strategies targeting CD73 or adenosine receptors have been used to restore immune surveillance and improve the therapeutic effect. For example, nanoparticles loaded with A2A receptor agonists can temporarily regulate the permeability of the blood-brain barrier, improve the delivery efficiency of chemotherapy drugs and immune antibodies, enhance the anti glioma immune response and reverse hypoxia related resistance (Meng et al., 2021), suggesting that targeting adenosine pathway can simultaneously play a dual therapeutic benefit at the level of tumor cells and immune microenvironment.

In addition, hypoxic tumor microenvironment can also affect the function of brain endothelial cells and the integrity of blood-brain barrier through adenosine signaling. Under hypoxia, the related factors secreted by glioma can damage the mitochondrial function of brain endothelial cells, leading to abnormal changes in barrier permeability, and then affect the drug penetration and therapeutic effect; The activation of adenosine receptors can directly participate in the regulation of endothelial cell function and tight junction integrity, and further affect the distribution and pharmacokinetics of drugs in tumor tissues (Rado et al., 2022). In general, adenosine signaling pathway, which starts from adenosine production mediated by CD73 and takes A2B and A3 receptors as the core, plays multiple roles in glioma hypoxia related drug resistance: it not only enhances the activity of multidrug resistance transporters, maintains the survival of tumor cells in hypoxia environment, but also shapes the immunosuppressive microenvironment, and aggravates drug resistance by affecting the blood-brain barrier. The intervention strategy targeting this pathway is expected to overcome drug resistance by improving intracellular drug retention, restoring anti-tumor immunity, optimizing drug delivery and other multiple effects. In the future, it is still necessary to further analyze its precise molecular mechanism, and develop efficient targeted treatment methods that adapt to the heterogeneous microenvironment of glioma (Rado et al., 2022).

### 4.3 Relationship between tumor associated immune cells and drug resistance

Tumor associated immune cells are the core of shaping the immunosuppressive microenvironment of glioma, in which tumor associated macrophages and regulatory T cells (Tregs) play a leading role, and ultimately promote tumor malignant progression and treatment resistance. Tumor associated macrophages (TAMs) in the glioma microenvironment tend to be M2-like phenotypes. These cells become an important driver of immune escape by secreting anti-inflammatory factors, mediating tissue remodeling and angiogenesis, and sheltering tumor cells from immune attack. Single cell RNA sequencing data confirmed that the infiltration of M2-like TAMs in recurrent gliomas was much higher than that in primary tumors, confirming its key role in tumor recurrence and chemotherapy resistance (You et al., 2023). Specifically, M2-like TAMs can activate the PI3K/Akt/HIF-1 α/CA9 signaling pathway in glioma cells through the interaction of SPP1-CD44, which can not only improve the malignant degree of tumor, but also induce strong immunosuppressive reaction, further aggravate drug resistance, and form a harsh microenvironment that hinders anti-tumor immunity and reduces the efficacy of conventional treatment.

As another kind of key immunosuppressive cells, regulatory T cells can further strengthen the immunosuppressive microenvironment by suppressing the activity of effector T cells and inducing tumor immune tolerance. The high infiltration of regulatory T cells in glioma microenvironment is closely related to the poor prognosis and low response to chemotherapy. In tumor tissues enriched with regulatory T cells, the expression of PD-L1 and other immune checkpoint molecules increased significantly, which further enhanced tumor immune escape and weakened the body’s ability to clear tumors (Song et al., 2025). Relevant studies have also found that *NUP37* molecule is positively correlated with tumor associated macrophages and regulatory T cell infiltration, and negatively correlated with anti-tumor immune cell infiltration such as CD8+T cells and NK cells, which fully proves that this highly immunosuppressive microenvironment is an important protective umbrella for tumor survival and drug resistance (He et al., 2022). It not only suppresses the immune-mediated tumor killing effect, but also helps tumor cells adapt to treatment stress.

**FIGURE 3:**
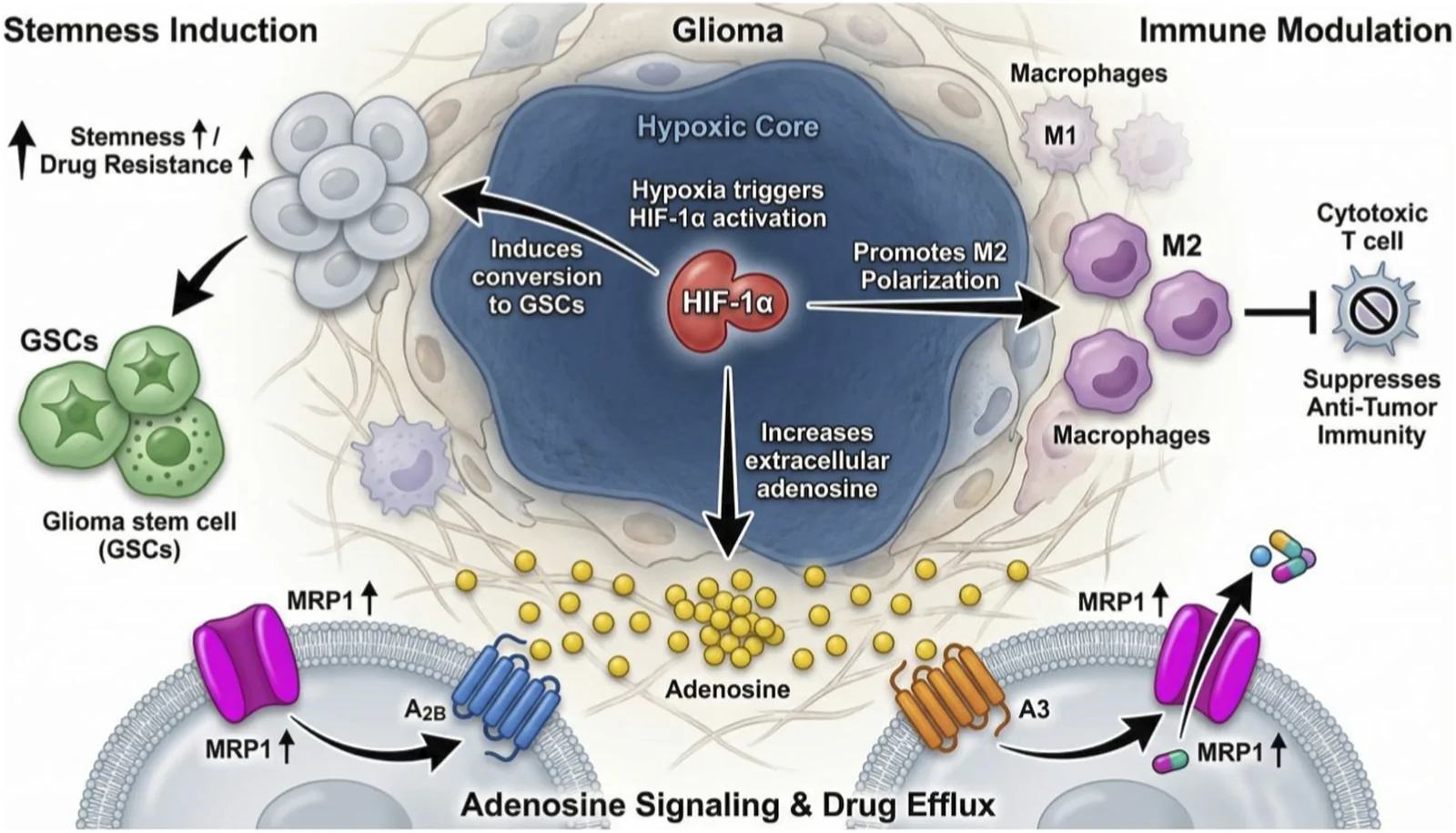
In the hypoxic core region, HIF-1α regulates glioma stemness, tumor microenvironment immune remodeling, and adenosine-mediated drug efflux resistance through multiple pathways.

The immunosuppressive microenvironment constructed by tumor associated macrophages and regulatory T cells can directly regulate the drug resistance pathway and reduce the efficacy of chemotherapy drugs. Glioma cells can directly obtain chemoresistance phenotype by up regulating the expression of drug efflux transporters and anti apoptotic proteins through the signal molecules secreted by TAMs. In addition, the exosomes secreted by glioma cells also play an important role in the remodeling of the immune microenvironment. These exosomes carry bioactive substances such as miRNA and protein, which can reprogram the function of immune cells and help tumor proliferation and treatment resistance (Guo et al., 2022; Luo et al., 2023). The exosomes communication between tumor cells and immune cells constitutes a complex regulatory network for promoting drug resistance. Moreover, glioma related molecules are also involved in the regulation of immune microenvironment. For example, CD99 molecule can promote the infiltration of immunosuppressive TAMs and enhance tumor adaptability under hypoxic environment. Its high expression is often accompanied by chemotherapy and radiotherapy resistance (Shang et al., 2023); L-lysine crotonylation related long-chain non coding RNA is also associated with M2 macrophages, increased regulatory T cell infiltration, and high expression of immune checkpoints, and is involved in the process of glioma chemotherapy resistance (Figure 3; Song et al., 2025).

According to the existing studies, tumor associated macrophages and regulatory T cells are the core cells that mediate glioma immune escape and chemotherapy resistance. The immunosuppressive network constructed by them is an important obstacle to the effective treatment of glioma. Reprogramming M2 type TAMs into M1 phenotype of anti-tumor and inhibiting the function of regulatory T cells are expected to restore the body’s anti-tumor immunity and enhance the sensitivity of chemotherapy. Since TAMs can induce immunosuppression and drug resistance by activating PI3K/Akt pathway (You et al., 2023; Dmello et al., 2023), the combination of immunomodulators and conventional chemotherapy can also break the immunosuppressive barrier and improve the therapeutic effect. However, the immune cells in the glioma microenvironment are highly heterogeneous and plastic. In order to overcome the problem of drug resistance, it is still necessary to further study the dynamic interaction mechanism of tumor immune cells and develop new treatment schemes that accurately target the immunosuppressive network.

## 5 Regulatory role of hippo signaling pathway in glioma drug resistance

### 5.1 Basic components and functions of Hippo pathway

Hippo signaling pathway is an evolutionarily highly conserved kinase cascade, which plays a fundamental physiological role in organ size maintenance, tissue homeostasis regulation and cell fate determination by accurately regulating the dynamic balance of cell proliferation and apoptosis. The core components of this pathway are composed of a series of kinases, mainly including Mst1/2 (mammalian Ste20 like protein kinase 1/2) and LATS1/2 (large tumor suppressor 1/2). Both of them realize the step-by-step regulation of pathway activity by phosphorylating downstream effectors in turn. The core foothold of pathway function lies in the strict regulation of transcription co activator Yap (yes related protein 1) and TAZ (transcription co activator containing PDZ binding motif). When Hippo pathway is normally activated, mst1/2 will be phosphorylated and *LATS1/2* will be further activated, thereby mediating the activity regulation of downstream YAP/TAZ (Masliantsev et al., 2021).

Once the Hippo pathway is inactivated, YAP/TAZ will undergo dephosphorylation modification, and then translocate into the nucleus, combining with the TEAD family transcription factors to start a specific gene expression program. Such procedures can not only effectively promote cell proliferation and inhibit cell apoptosis, but also widely participate in physiological processes such as organ growth and tissue regeneration. This regulatory axis is not only crucial in the development stage of the body, but also plays a key role in the maintenance of adult tissue homeostasis and damage repair. A large number of studies have confirmed that the abnormal regulation of Hippo signaling pathway, especially the over activation of YAP/TAZ, is closely related to the occurrence and development of a variety of malignant tumors, and is an important inducement for malignant transformation of tumors (Masliantsev et al., 2021; Wang et al., 2019).

The kinase cascade of Hippo pathway does not work in isolation, but is strictly regulated by a variety of upstream signals, mainly including intercellular contact, mechanical signals and various extracellular signals. Stripak complex, SAV1, MOB1 and other scaffold proteins can regulate the kinase activity of Mst1/2 and LATS1/2, integrate a variety of upstream signal inputs, and achieve fine regulation of pathway output. Recent studies have also found that biomolecular aggregates formed by phase separation play a key role in the spatio-temporal regulation of the core components of Hippo pathway, adding a new level of apparent regulation to the pathway regulation, and further highlighting the complexity and adaptability of the pathway in response to changes in the cell microenvironment (Shao et al., 2025; Wang L. et al., 2022).

From the perspective of biological function, Hippo signaling pathway accurately controls the key life processes such as cell proliferation, apoptosis, differentiation and organ size, and plays an anti-tumor role by inhibiting the abnormal activation of YAP/TAZ to prevent excessive cell proliferation. On the contrary, when Hippo pathway conduction is damaged, YAP/TAZ will lose strict control, and its continuous activation will lead to abnormal cell proliferation, apoptosis resistance, and ultimately promote the malignant progression of tumor. In gliomas, abnormal expression or dysfunction of the core components of Hippo pathway is closely related to enhanced tumor invasiveness, poor prognosis and chemoradiotherapy resistance (Masliantsev et al., 2021; Aguennouz et al., 2020). In addition, Hippo pathway is also involved in the regulation of tumor immune microenvironment, cell aging and other processes, thereby affecting tissue homeostasis and disease development; At the same time, this pathway can also interact with Wnt/β-catenin and other carcinogenic pathways, which further expands its functional spectrum in the malignant progression of tumors. It not only regulates the internal biological process of cells, but also participates in shaping the tumor microenvironment, which is crucial for the occurrence and development of glioma (Guo et al., 2021; Chen Q. et al., 2025).

To sum up, Hippo pathway, with *MST1/2* and *LATS1/2* kinases as the core, forms a complete kinase cascade reaction system, accurately regulates the nuclear translocation and transcriptional activity of YAP/TAZ, and becomes a key molecular switch to maintain tissue homeostasis by regulating cell proliferation, apoptosis and organ size, and also plays an important role in the occurrence and development of malignant tumors such as glioma. The upstream regulation mechanism of this pathway is complex and diverse, and has high clinical targeting value (Masliantsev et al., 2021; Shao et al., 2025). It is worth noting that the activity of Yap/TAZ is dynamically regulated by phosphorylation and subcellular localization, which also means that the treatment strategy targeting Yap/TAZ axis can effectively inhibit the proliferation of glioma and reverse drug resistance, providing a promising direction for basic research and clinical intervention of glioma.

### 5.2 Dysfunction of the Hippo pathway and glioma progression

In glioma, the abnormality of Hippo signaling pathway has been proved to be the core factor driving tumor occurrence and progression. The pathway core kinases *MST1/2* and *LATS1/2* can inhibit the nuclear translocation process of YAP and TAZ through phosphorylation modification. Once the pathway signal is blocked, YAP/TAZ will accumulate in a large number in the nucleus and activate the target genes that promote proliferation and survival in combination with the transcription factors of the TEAD family. This abnormal activation directly promotes the malignant progression of glioma and induces treatment resistance, which fully reflects the carcinogenic effect of this pathway in brain tumors (Masliantsev et al., 2021). The proteomic study of glioblastoma also identified the key pathway proteins such as PP1 γ and Yap1, whose high expression was closely related to the high grade of tumor and the poor prognosis of patients. The expression of PP1 γ was positively correlated with Yap1 and Sox2, while the low expression of PP1 γ was correlated with the favorable IDH1 mutation status in low-grade gliomas, suggesting that the PP1 γ - Yap1 axis was out of balance, which could be used as a prognostic marker and potential therapeutic target for gliomas (Xue et al., 2020).

Hippo pathway is also finely regulated by non coding RNA, which further aggravates the complexity of pathway imbalance in glioma. MicroRNAs such as miR-221 and miR-10b can directly target LATS1, the core kinase of the pathway, inhibit its expression, thereby reducing the phosphorylation level of Yap, enhancing the nuclear localization of Yap, and accelerating the proliferation of glioma cells. Conversely, knocking down the above microRNAs can inhibit tumor growth, confirming the key role of non coding RNA in pathway deregulation (Aguennouz et al., 2020). In addition, long non coding RNAs are also involved in the regulation of pathways. For example, *lncRNA* *MKLN1-AS* can combine with mir-126-5p to activate the Hippo pathway mediated by TEAD1 and boost the occurrence and development of glioma. Knocking down this *lncRNA* can inhibit cell proliferation and angiogenesis (Chen S. et al., 2025); Epigenetic modification is also involved. In IDH mutant low-grade glioma, the abnormal methylation of LATS2 promoter is also closely related to the prognosis of patients (Gu et al., 2020).

Hippo pathway does not play an independent role. It interacts with PI3K/Akt, Wnt/β-catenin and other carcinogenic pathways, which together constitute the regulatory network of malignant progression of glioma. Transcription factor p75cux1 can promote glioma invasion by activating β-catenin mediated epithelial mesenchymal transition, and regulate hippo and PI3K/Akt pathways, confirming the close association between these pathways (Xu A. et al., 2021); While tankyrase, which targets the Wnt/β-catenin and hippo pathways at the same time, can not only inhibit the proliferation of glioma stem cells, but also enhance the sensitivity of tumors to temozolomide, suggesting that the combination of targeted cross pathways can effectively overcome the treatment resistance (Kierulf-vieira et al., 2020). From the perspective of treatment, targeting Hippo pathway has high clinical potential. Viteporfin can block the interaction of YAP/TAZ-TEAD. Small molecule inhibitors such as xmu-mp-1 have also shown good efficacy in preclinical studies. Some drugs, such as imipramine, can also cooperate with temozolomide to exert anti-tumor effect, providing a new idea for the treatment of drug-resistant glioma (Wang et al., 2021c).

In conclusion, the disorder of Hippo pathway, especially the over activation of Yap/TAZ and its abnormal combination with TEAD transcription factors, is the key inducement for the occurrence, development and treatment resistance of glioma, and the cross interaction between pathways further amplifies its carcinogenic effect. At present, the treatment strategies for Hippo pathway mainly focus on the direct inhibition of Yap/TAZ, the intervention of upstream regulatory factors, epigenetics and non coding RNA targeting. In the future, it is still necessary to further analyze the precise molecular mechanism of Hippo pathway imbalance in different glioma subtypes, so as to optimize the targeted therapy and ultimately improve the clinical prognosis of glioma patients.

### 5.3 Hippo pathway and tumor immunosuppression

Hippo signaling pathway plays a central role in tumor immunosuppression and immune escape, and its regulatory effect is mainly achieved by downstream key effector yes related protein (Yap) and transcription co activator (TAZ) with PDZ binding motif. As a core transcription co activator, Yap/TAZ can provide a molecular basis for tumor immune escape by precisely regulating the expression of a variety of immune checkpoint molecules and immunosuppressive factors. Previous studies have confirmed that Yap activation can directly upregulate the expression of “do not eat me” signal molecule CD24, and then inhibit macrophage mediated phagocytosis, clearly revealing the direct mechanism of Hippo pathway effector molecules promoting immune escape (Zhou et al., 2024); In lung adenocarcinoma, hippo/Yap axis can regulate the expression of PD-L1. Inhibition of Yap activity can significantly reduce the level of PD-L1 and enhance the anti-tumor immune response, further confirming the core position of Yap as a key regulatory factor of immune checkpoint (Li L. et al., 2023). These studies together show that Yap/TAZ can construct a tumor microenvironment (TME) with strong immunosuppressive properties by regulating the expression of immune checkpoint molecules, providing support for tumor escape from immune surveillance.

The regulatory effect of Hippo pathway components is not only limited to immune checkpoint molecules, but also widely affects the composition and function of immune cells and stromal cells in tumor microenvironment. The abnormal activation of Yap/TAZ is closely related to the increased infiltration and polarization of tumor associated macrophages (TAMs), especially promoting the transformation of TAMs to M2-like immunosuppressive phenotype, which can significantly promote the malignant progression of tumors and inhibit the body’s anti-tumor immune response. It can be seen that Hippo Yap signaling pathway can reshape the tumor immune microenvironment by regulating TAMs activity (Yang et al., 2020). In addition, the disorder of Hippo pathway in tumor cells can also promote the formation of immunosuppressive cancer-related fibroblasts (CAFs), such as ncam1+α sma + CAFs secreting transforming growth factor - β (TGF - β), which can induce T cell inactivation and further aggravate immune escape (Thinyakul et al., 2024). The dual regulation of Hippo signal on stromal cells and immune cells jointly constructs a highly inhibitory tumor microenvironment, significantly weakening the body’s effective anti-tumor immune response.

In addition to regulating immunosuppressive cells, Hippo signaling pathway can also directly affect the biological function of T cells. Yap, as a key immunosuppressive factor, can inhibit the activation, differentiation and effector function of T cells. Studies have found that the deletion of Yap in T cells can significantly enhance its activation ability and tumor invasion ability, and then improve the anti-tumor immune efficacy *in vivo* (Stampouloglou et al., 2020). It is worth noting that the regulation mechanism of Hippo pathway on T cell homeostasis is complex and context dependent, including both the classical YAP/TAZ dependent regulation mode and the regulation pathway that does not depend on the core effector molecule, and the effects of these mechanisms on different T cell subsets are significantly different (Bouchard et al., 2020). This dual regulatory feature suggests that targeting Hippo pathway components is expected to restore the normal function of T cells, providing a new entry point for improving the effect of immunotherapy. At the same time, the immunosuppressive effect of Hippo signaling pathway can be further confirmed by reshaping the tumor microenvironment to resist immune checkpoint blocking (ICB) therapy: YAP/TAZ activation can not only upregulate the PD-L1 expression of tumor cells themselves, but also promote the PD-L1 expression of CAFs and other immunosuppressive cells, thereby enhancing tumor resistance to ICB therapy (Kim and Nam, 2025); The combination of YAP/TAZ inhibition and immune checkpoint inhibitors has shown good effects in preclinical studies, which can significantly enhance the tumor clearance ability mediated by T cells and effectively overcome the drug resistance mechanism (Zhang and Zhan, 2025), which highlights the great potential of targeting Hippo pathway to regulate the immunosuppressive microenvironment and improve the effect of immunotherapy.

In low-grade gliomas, the disorder of Hippo signaling pathway is also closely related to the immunosuppressive microenvironment, which is typically characterized by increased infiltration of myeloid derived suppressor cells (MDSCs) and regulatory T cells, further weakening the body’s effective anti-tumor immune response (Guo et al., 2023); At the same time, the activation mode of Hippo pathway is closely related to the immune cell infiltration characteristics of low-grade glioma and the clinical prognosis of patients, which provides an important basis for the formulation of personalized treatment strategies (Guo et al., 2023). In summary, Hippo pathway and its effector Yap/TAZ regulate tumor immunosuppression through multi-dimensional mechanisms, including up regulating immune checkpoint molecules such as PD-L1 and CD24, regulating the infiltration and polarization of immunosuppressive cells such as TAMs and CAFs, and directly inhibiting the activation and function of T cells, which fully highlights its core position as the main regulator of tumor immune escape, and also provides a key target for improving the effect of immunotherapy for glioma and other cancers. Future research should focus on clarifying the environmental dependent effect of Hippo signal in different immune cell populations within the tumor microenvironment, and provide theoretical support for precise targeted intervention (Kim and Nam, 2025; Zhang and Zhan, 2025; Guo et al., 2023).

### 5.4 Therapeutic strategies for Hippo pathway

As a highly conserved regulator of organ size and tissue homeostasis, Hippo signaling pathway plays a key role in the pathogenesis and treatment resistance of glioma. Its core kinase cascade reaction (including *MST1/2* and *LATS1/2*) can inhibit the carcinogenic activity of YAP and TAZ by preventing the nuclear translocation of transcription co activators and their subsequent interaction with tea transcription factors. The disorder of this pathway is closely related to the progression of glioma, making it a potential target for new therapeutic intervention (Masliantsev et al., 2021). The use of small molecule inhibitors to target the regulation of Yap/TAZ activity is a feasible strategy to curb the progression of glioma. Viteporfin, a drug inhibitor, can significantly inhibit the growth of tumor cells in the patient derived glioblastoma stem cell culture system by blocking the interaction of Yap/taz-TEAD. It is worth noting that the inhibition of YAP/TAZ by viteporfin can reduce cell proliferation, while the specific inhibition of TEAD3 will change the metabolic pathways such as sterol and cholesterol biosynthesis (Masliantsev et al., 2025). These findings highlight that Hippo effector molecules play a multidimensional role not only in regulating cell proliferation, but also in maintaining the key metabolic process of glioma.

In addition to directly targeting Yap/TAZ, small molecule inhibitors that regulate the upstream components of Hippo pathway also show potential in glioma treatment, but the complexity of pathway regulation determines the necessity of precise targeting. The mst1/2 kinase inhibitor xmu-mp-1 instead promotes the proliferation of low-grade glioma cells (Guo et al., 2023), while tankyrase inhibitors (such as g007-lk) that simultaneously act on Wnt/β-catenin and Hippo pathway can inhibit the proliferation of glioma stem cells and make cells sensitive to temozolomide, a standard chemotherapy drug for glioma, suggesting that such inhibitors may enhance the efficacy of temozolomide in glioblastoma models (Kierulf-vieira et al., 2020). In addition, tricyclic antidepressant imipramine was found to inhibit Yap activity independently of the classical Hippo pathway, promote the translocation of Yap cytoplasm and enhance the efficacy of temozolomide (TMZ) in glioma models. This combination therapy shows a synergistic tumor growth inhibition effect, highlighting the potential of reusable drugs that play a role through the non Hippo pathway (Wang et al., 2021c).

Immunotherapy combined with Hippo pathway regulation provides another promising way to overcome the drug resistance of glioma. The immunosuppressive tumor microenvironment in glioblastoma is partially regulated by the imbalance of Hippo pathway, which will promote chemotherapy resistance and immune escape (Casati et al., 2021). Recent preclinical studies have shown that the combination of radiotherapy and immunotherapy (such as CSF-1R inhibition) can overcome the radiotherapy resistance of glioma models. Transcriptome analysis shows that Hippo pathway genes are enriched in treatment resistant tumors, and the gene characteristics of Hippo components are related to the prognosis and treatment response of patients. These findings highlight Hippo pathway as a potential molecular target to enhance anti-tumor immunity and make glioma sensitive to conventional treatment (Zhang et al., 2024). In addition, glioma molecular subtypes based on machine learning and hippo/YAP pathway activation spectrum have identified patient subgroups with different immune infiltration patterns and drug sensitivity, providing an important basis for personalized treatment (Guo et al., 2023).

**FIGURE 4:**
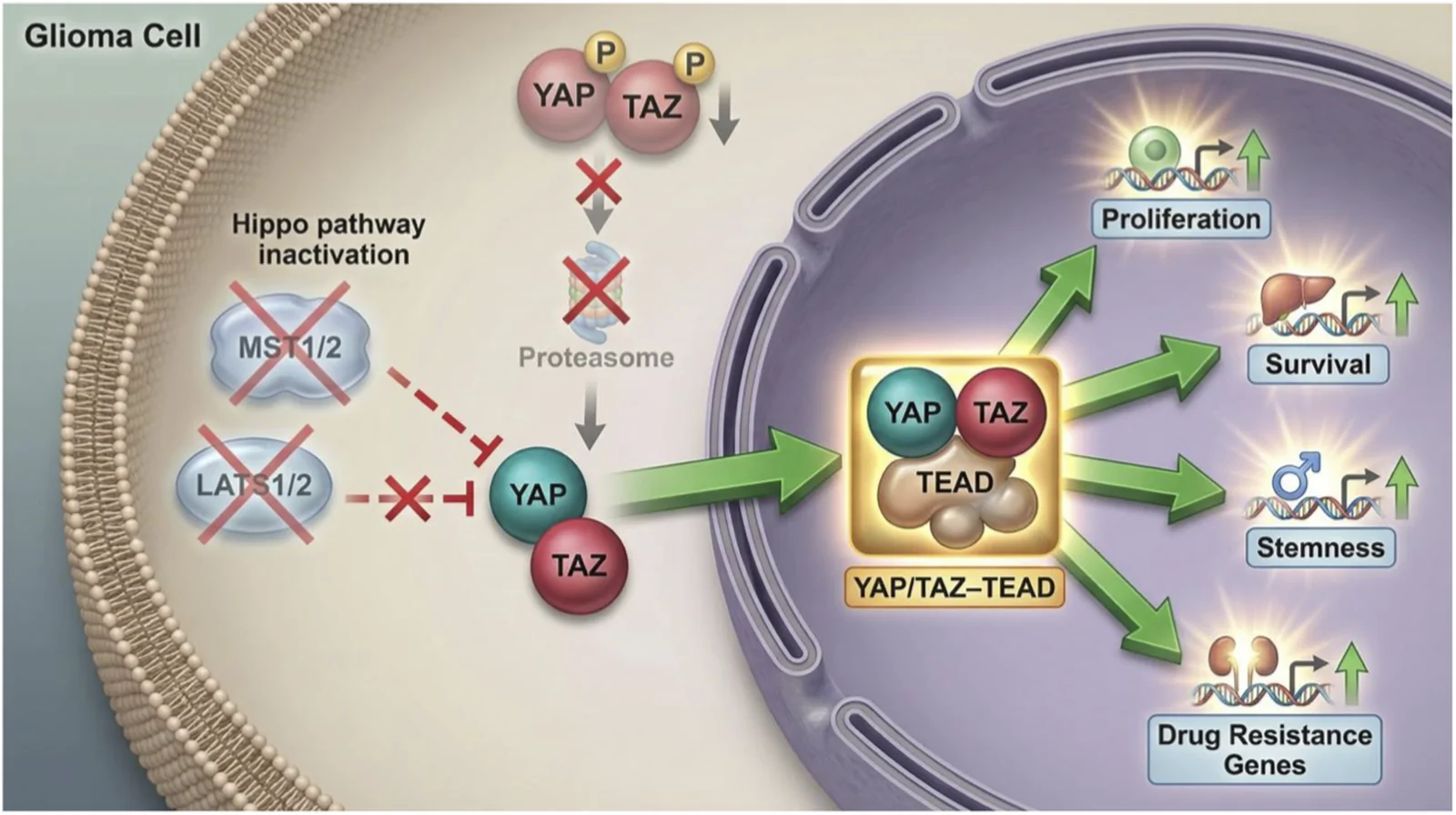
After the inactivation of the Hippo pathway, YAP/TAZ enters the nucleus and binds to TEAD, promoting the expression of genes related to glioma proliferation, survival, stemness, and drug resistance.

The regulatory network of Hippo pathway in glioma is more complex due to the participation of non coding RNA and ubiquitin proteasome system components, which can precisely regulate the stability and activity of Yap/TAZ. For example, long non coding RNA *MKLN1-AS* can promote glioma progression by activating mir-126-5p/TEAD1 axis (Chen S. et al., 2025); Similarly, ubiquitin ligases such as WWP2 can affect the phosphorylation and activity of Yap by regulating the ubiquitination and degradation of the core Hippo kinase LATS2, suggesting that the post-translational modification of Hippo pathway components may affect glioma progression (Fan and Zou, 2024). In view of the complex interactions among signal transduction, epigenetics and metabolic factors regulated by Hippo pathway, the combination therapy of small molecule inhibitors, immunotherapy and chemotherapy has the most prospect for overcoming glioma drug resistance and improving the survival rate of patients (Figure 4).

In conclusion, small molecule inhibitors targeting YAP/TAZ activity, such as viteporfin and tankyrase inhibitors, have shown efficacy in preclinical glioma models and often enhance the effect of standard chemotherapy. The combination of Hippo pathway regulation and immunotherapy strategy can solve the specific immunosuppressive microenvironment problem of glioma, and may overcome the treatment resistance. With the deepening understanding of the molecular characteristics of the Hippo pathway regulated by non coding RNA and ubiquitin mechanism, the scope of potential therapeutic targets has been further expanded. These multi-dimensional treatment strategies highlight the core position of Hippo pathway in glioma biology, making it an important potential target for future clinical intervention.

## 6 Non-coding RNA-mediated mechanisms of glioma drug resistance

### 6.1 Regulation of microRNA in drug resistance

MicroRNAs (miRNAs) are a class of small non coding RNAs, which mainly regulate gene expression through post transcription, and have become key regulatory factors in the study of glioma drug resistance. Its regulatory effect is widely involved in the expression regulation of multidrug resistance transporters, apoptosis related genes and stem cell markers. These mechanisms are intertwined and together constitute the complex pathological basis of glioma treatment failure. Among them, carcinogenic miRNA clusters such as miR-21 and miR-125b have been confirmed by many studies to promote the development of glioma drug resistance by regulating multiple cellular pathways (Jegathesan et al., 2024).

One of the key mechanisms of miRNA affecting glioma drug resistance is through regulating the expression of ATP binding cassette (ABC) family drug efflux transporters. The abnormal activation of these transporters will lead to the reduction of the concentration of chemotherapeutic drugs in cells, which will lead to drug resistance. MiRNA can reverse this process by targeting this family of proteins. For example, mir-9-5p can directly target ABC1 (member 1 of ATP binding box C subfamily), significantly reduce its expression level, thereby enhancing the sensitivity of glioma cells to temozolomide, a first-line chemotherapy drug (Chen et al., 2021); Similarly, mir-7-5p enhances the chemosensitivity of glioma cells and promotes tumor cell apoptosis by down regulating the expression of key multidrug resistance (MDR) proteins (Gajda et al., 2020), which fully reflects the important potential of miRNA in the regulation of drug efflux mechanism.

In addition to regulating drug transporters, miRNAs can also affect the survival ability of glioma cells under chemotherapy stress by regulating apoptosis related genes, and then participate in the regulation of drug resistance. The expression level of apoptosis related genes directly determines the effect of chemotherapy, and miRNA can break the balance between cell death and survival by targeting these genes. For example, mir-526b-3p can effectively inhibit the resistance of glioma cells to adriamycin, promote tumor cell apoptosis and inhibit the proliferation of drug-resistant cells by targeting MAPK ε 1 (Qiu et al., 2022); Mir-1246 is closely related to the poor prognosis of glioma patients treated with temozolomide (TMZ) by targeting *CCNG2* gene involved in cell cycle regulation and apoptosis (Wang et al., 2021d). In addition, miRNA can also regulate the stem characteristics of glioma stem cells, which is the key inducement of glioma drug resistance and recurrence. Glioma initiating cells have significant tolerance to chemotherapy drugs by virtue of their stem cell characteristics and the ability to escape immune apoptosis. Studies have shown that the delivery of miR-34a through customized lipoprotein like nanostructures can effectively inhibit the expression of stem cell markers such as Sox2 and Notch1 in glioma stem cells, reduce chemotherapy resistance and prolong the survival time of glioma models (Jiang et al., 2020), highlighting the important role of miRNA in targeting drug-resistant stem cell populations.

Among the many miRNAs involved in the regulation of glioma drug resistance, miR-21, as one of the most concerned oncogenic miRNAs, widely promotes the development of drug resistance by regulating apoptosis inhibition, promoting cell proliferation and remodeling the tumor microenvironment. Its high expression level is closely related to the high grade of glioma and the poor prognosis of patients, and has become a potential therapeutic target (Aloizou et al., 2020); Another consistent oncogenic miRNA, miR-125b, has also been confirmed to promote the formation of drug resistance in high-grade glioma (Jegathesan et al., 2024). It is worth noting that these miRNAs do not play a role in isolation, but often cooperate with long-chain non coding RNA to form a complex regulatory network and accurately regulate the expression of drug resistance related genes (Wu and Qian, 2019). In general, the regulatory role of miRNA in glioma drug resistance has multidimensional characteristics. In addition to the above mechanisms, its regulation of key signaling pathways such as Wnt/β-catenin further highlights its core position in the formation of drug resistance (Tabnak et al., 2021; Jia et al., 2022). Targeting specific miRNAs or their regulatory networks is expected to effectively overcome the drug resistance of glioma and improve the therapeutic effect. In view of the high heterogeneity of glioma and the diverse functions of miRNA, the use of individualized treatment strategies regulated by miRNA may become a necessary means to achieve the best clinical outcomes, such as the differential expression of miR-21 and miR-125b in different glioma subtypes, suggesting that customized intervention measures based on miRNA can enhance the sensitivity of specific patient groups to chemotherapy drugs (Jegathesan et al., 2024).

### 6.2 Long non coding RNA and drug resistance

Long non coding RNA (*lncRNA*) is a class of transcripts with a length of more than 200 nucleotides and no protein coding ability. By regulating gene expression and chromatin remodeling, *lncRNA* has become a key regulatory factor in glioma drug resistance. Its functions are widely involved in cell proliferation, migration, apoptosis, drug sensitivity and other cellular processes. The imbalance of *lncRNA* expression in glioma is deeply involved in the complex mechanism of drug resistance, especially in the regulation of drug resistance of temozolomide (TMZ), a first-line chemotherapy drug for glioma.

One of the key mechanisms of *lncRNA* mediated glioma drug resistance is that it acts as a competitive endogenous RNA (Cerna) to adsorb microRNA (miRNA), and then regulates the expression of miRNA target genes involved in carcinogenic signaling pathway. For example, *lncRNA* *MALAT1* is overexpressed in glioma, which is closely related to tumor growth, metastasis, apoptosis and chemotherapy resistance, and is an important molecule to promote glioma progression and treatment resistance (Ji et al., 2024; Li H. et al., 2023); Similarly, the expression of *lncRNA* *DLEU1* is upregulated in glioma tissues, which promotes the proliferation and migration of tumor cells by regulating cyclins and epithelial mesenchymal transition (EMT) markers. Knockdown of *DLEU1* can enhance the sensitivity of glioma cells to TMZ by inhibiting autophagy and inducing apoptosis, highlighting its potential value as a target to overcome chemotherapy resistance (Lv et al., 2020).

In addition, *lncRNA* can participate in the formation of chemotherapy and radiotherapy resistance by regulating the stemness of glioma stem cells, and the stemness of glioma stem cells is the key inducement of tumor resistance and recurrence. For example, *lncRNA* *GSCAR* can stabilize the expression of stem cell marker Sox2 and maintain the stemness of glioma stem cells through the positive feedback loop involving *DHX9* and IGF2BP2 protein. The deletion of *GSCAR* can significantly inhibit the proliferation of tumor cells and enhance the sensitivity of glioma cells to TMZ (Jiang et al., 2023); Another *lncRNA*, *NONHSAT141192.2*, increased the expression of Sox2 and *PIK3R3* by adsorbing mir-4279, promoted the proliferation of glioma stem cells and enhanced radiation resistance, which further confirmed that *lncRNA* played a wide role in a variety of treatment resistance by regulating the characteristics of stem cells (Wang et al., 2025).

Epigenetic regulation is also an important way of *lncRNA* mediated glioma drug resistance. Among them, *lncRNA* related to RNA methylation (such as rp11-98i9.4 and rp11-752g15.8) can effectively inhibit the proliferation of glioma cells and TMZ resistance (Huang et al., 2023); In addition, *lncRNA* RMRP and Wnt/β - catenin signaling pathway form a positive feedback loop, which promotes TMZ resistance by down regulating the negative regulator *ZNRF3* of this pathway. Targeting this pathway can block the continuous activation of Wnt/β-catenin, thereby improving the therapeutic effect of TMZ (Liu et al., 2021). At the same time, *lncRNA* can also affect the drug sensitivity of glioma cells by regulating DNA repair pathway and cell apoptosis, which are the key factors determining the tumor response to chemotherapy drugs. For example, *lncRNA* UCA1 can upregulate the expression of DNA repair enzyme MGMT by adsorbing mir-182-5p and enhance TMZ resistance, while knockdown of UCA1 or overexpression of mir-182-5p can reduce the level of MGMT and make glioma cells more sensitive to TMZ induced apoptosis (Cheng et al., 2022); TGF - β 1-induced *lncRNA* H19 and *HOXD-AS2*, through competitive binding with the splicing factor ksrp (ksrp is essential for the biosynthesis of mir-198), reduce the level of mir-198 and increase the expression of *MGMT*, ultimately leading to TMZ resistance, revealing the synergistic regulation of *lncRNA*, miRNA and DNA repair mechanism in glioma chemotherapy resistance (Nie et al., 2021).

The role of *lncRNA* in the regulation of drug resistance is not limited to TMZ chemotherapy, but also widely involved in the regulation of resistance to platinum drugs, radiotherapy and other treatments. For example, *LINC00470* inhibits autophagy and enhances cisplatin resistance by inhibiting PTEN expression; *LINC01123* can upregulate *CENPB* by adsorbing mir-151a, promote the radiation resistance of glioma cells, and enhance their survival ability in the radiation environment (Chen et al., 2023; Tian et al., 2022). In addition, some *lncRNAs* can further promote multidrug resistance and tumor invasiveness by regulating the expression of drug efflux transporters and EMT markers. For example, *LINC00883* adsorbs mir-136 and upregulates NEK1 through sponge, resulting in increased expression of multidrug resistance related proteins and decreased apoptosis, thus enhancing drug resistance (Li and Gao, 2021); *NCK1-AS1* promotes glioma cell proliferation, radiation resistance and chemotherapy resistance through mir-22-3p/*IGF1R* Cerna axis (Wang B. et al., 2020).

In summary, *lncRNAs* form a complex regulatory network by regulating gene expression, signaling pathways, epigenetic modifications and cell phenotypes, which jointly drive the treatment resistance of glioma. Targeting specific *lncRNAs* or their interacting partners provides new ideas and directions for overcoming the drug resistance of glioma treatment and improving the clinical prognosis of patients. Future research should focus on verifying these potential therapeutic targets in the clinical environment, and explore the combined therapy of *lncRNA* regulation, so as to further improve the therapeutic effect of glioma.

### 6.3 Role of cyclic RNA in drug resistance of glioma

Circular RNA (*circRNA*) is a kind of key non coding RNA molecules with covalently closed circular structure, which gives it significant stability and anti exonuclease ability. In glioma, the role of *circRNA* in the regulation of tumor resistance (especially to temozolomide (TMZ), a first-line chemotherapy drug for glioma), has attracted increasing attention, and has become a research hotspot in the field of glioma resistance regulation. One of the main mechanisms of *circRNA* affecting the drug resistance of glioma is that it acts as a microRNA (miRNA) sponge, which indirectly regulates the expression of miRNA target genes involved in the drug resistance pathway by specifically isolating miRNA, and then participates in the formation of drug resistance phenotype. For example, hsa_circ_0110757 has been confirmed to promote the resistance of glioma cells to TMZ by isolating hsa-mir-1298-5p, leading to the upregulation of the expression of target gene *ITGA1*, thereby inhibiting the apoptosis of glioma cells and promoting tumor invasion (Li H. et al., 2021); Similarly, *cricHIPK3* regulates TMZ resistance by targeting mir-524-5p, regulating *KIF2A* expression and PI3K/Akt signaling pathway (Yin and Cui, 2021). These findings suggest that *circRNA* can play a regulatory role by competitively binding miRNA, relieve the inhibition of carcinogenic mRNA, activate the downstream pro drug resistance signal cascade, and promote the occurrence of glioma treatment resistance.

In addition to the core mechanism of miRNA sponge effect, *circRNA* can also participate in the regulation of drug resistance by directly regulating the key signaling pathways related to glioma drug resistance. PI3K/Akt pathway, as the core medium regulating cell proliferation, survival and metabolism, is often precisely regulated by *circRNA* in glioma cells. For example, *circSPECC1* regulated by Y-box binding protein-1 (YB-1) can capture mir-615-5p through the sponge effect, promote the growth of glioma cells, and then upregulate Huntington protein interacting protein-1 (Hip1) and activate *HIP1/AKT* pathway, participating in the regulation of drug resistance (Lan et al., 2023). In addition, *circRNA* can also affect other key pathways such as wnt/β - catenin signaling axis. For example, *circRNA* 0055412 regulates the activation of wnt/β - catenin signaling pathway by stabilizing *CAPG* mRNA, enhancing the resistance of glioma cells to cisplatin, highlighting the role of *circRNA* in the regulation of multidrug resistance beyond TMZ (Zhou Q. et al., 2022). These complex regulatory networks reveal that *circRNA*, as a node regulator, can integrate multiple drug resistance related pathways and participate in the regulation of glioma treatment response.

Exosome mediated *circRNA* transfer further magnifies its regulatory effect on glioma drug resistance, which is mainly achieved by promoting intercellular communication in the tumor microenvironment. Specific *circRNAs* are often enriched in the exosomes derived from drug-resistant glioma cells. These *circRNAs* can be transported from drug-resistant cells to sensitive cells, endowing sensitive cells with drug-resistant phenotypes, and accelerating the spread of drug resistance in tumor populations. For example, *circWDR62* is enriched in the exosomes of TMZ resistant glioma cells, which upregulates the expression of DNA repair enzyme MGMT by adsorbing mir-370-3p, and *MGMT* can effectively resist TMZ induced DNA damage, thus promoting chemotherapy resistance (Geng et al., 2022); Similarly, the exosome circ_0072083 regulates the expression of *NANOG* through the mir-1252-5p-mediated pathway, which involves *ALKBH5* dependent demethylation and ultimately enhances the resistance of glioma cells to TMZ (Ding et al., 2021). These observations indicate that *circRNA* can not only play a regulatory role in cells, but also play a paracrine effect through exosome transport, promoting the heterogeneity and adaptability of glioma cells under the pressure of chemotherapy, and further aggravating drug resistance.

In addition, *circRNA* can also participate in the regulation of glioma drug resistance through the interaction with other non coding RNAs and protein factors, and affect key cell processes such as apoptosis, autophagy and epithelial mesenchymal transition (EMT). For example, circ_0008344 regulates the expression of *RNF2* by binding mir-433-3p, thereby affecting the proliferation and apoptosis of glioma cells and regulating their radiosensitivity (Di et al., 2021). This interaction of multiple molecules and processes highlights the role of *circRNA* as a multifunctional regulator of drug resistance in glioma treatment, which can coordinate the complex molecular network that determines the fate of cells after treatment.

Based on the existing evidence, *circRNA* mainly promotes glioma drug resistance through two core mechanisms: first, as a miRNA sponge, it regulates the expression of key drug resistance related genes; Second, it directly participates in the regulation of drug resistance by regulating the core signaling pathways such as PI3K/Akt, Wnt/β-catenin and DNA repair mechanism. At the same time, exosome mediated *circRNA* transfer further enhanced its functional effect and promoted the diffusion of drug resistance characteristics in the tumor microenvironment. These multidimensional regulatory effects make *circRNA* a potential biomarker for the prognosis of glioma and a potential therapeutic target for overcoming drug resistance. Targeting the *circRNA* mediated regulatory network may provide a new strategy for improving the sensitivity of glioma cells to chemotherapy and improving the clinical prognosis of patients (Fan et al., 2023b; Wu et al., 2025). In view of the diversity of *circRNA* functions and its wide participation in multiple drug resistance mechanisms, future research should focus on clarifying the specific *circRNA* miRNA mRNA regulatory axis under different glioma subtypes and treatment background, so as to optimize the targeted intervention scheme and improve the treatment effect.

### 6.4 Potential of non coding RNAs as therapeutic targets

Non coding RNA (ncRNA), which includes long-chain non coding RNA, microRNA and circular RNA, has become a key molecule in regulating the pathogenesis, tumor progression and drug resistance of glioma. It has cell type specific expression characteristics and diverse regulatory functions, which makes it an ideal candidate for glioma targeted therapy. At present, a promising treatment strategy is to use antisense oligonucleotides (ASOs), miRNA mimics or inhibitors to precisely regulate the expression or activity of ncRNA related to drug resistance, so as to achieve targeted intervention on glioma. For example, through CRISPR interference (CRISPRi) screening, it was found that long non coding RNA *LNCRS-1* (a long non coding RNA conserved in the nucleus of primates) can be used as a glioma specific radiosensitizer. The antisense oligonucleotide targeting this *lncRNA* can selectively inhibit the growth of glioma cells and enhance their radiosensitivity, and has no significant effect on normal brain cells, which fully proves the feasibility and safety of ncRNA targeted intervention (Liu et al., 2020); Similarly, miR-21, as a classic carcinogenic miRNA highly expressed in glioma, is effectively targeted by CRISPR CAS gene knockout strategy. The results show that this strategy can significantly reduce the proliferation, migration and invasion of glioma cells *in vitro* and in mice with normal immune function, further suggesting its great potential as a therapeutic target (Nieland et al., 2022).

Although ncRNA targeted therapy has made significant progress in basic research, such therapeutic drugs still face many major challenges in the process of clinical transformation. The key is how to achieve efficient drug penetration through the blood-brain barrier and achieve tumor specific targeting to reduce the off target effect. The blood-brain barrier has strict permeability restrictions on most macromolecular substances (including nucleic acid drugs), which requires the development of innovative delivery systems to break through the barrier. At present, nanoparticles, exosomes or virus vectors modified to cross the blood-brain barrier are all potential delivery strategies. It is worth noting that ncRNA derived from exosomes is a natural carrier to promote intercellular communication in the glioma tumor microenvironment, and transforming exosomes to deliver therapeutic ncRNA or its inhibitors has become a promising direction to overcome delivery barriers and improve treatment efficiency (Jin and Bai, 2025; Marangon and Lecca, 2023). In addition, the high heterogeneity of glioma cells and immunosuppressive tumor microenvironment (TME) further increase the complexity of therapeutic effect, and also highlight the necessity of combining ncRNA targeted therapy with immunotherapy to construct a combined treatment strategy (Riyas Mohamed and Yaqinuddin, 2025).

**FIGURE 5:**
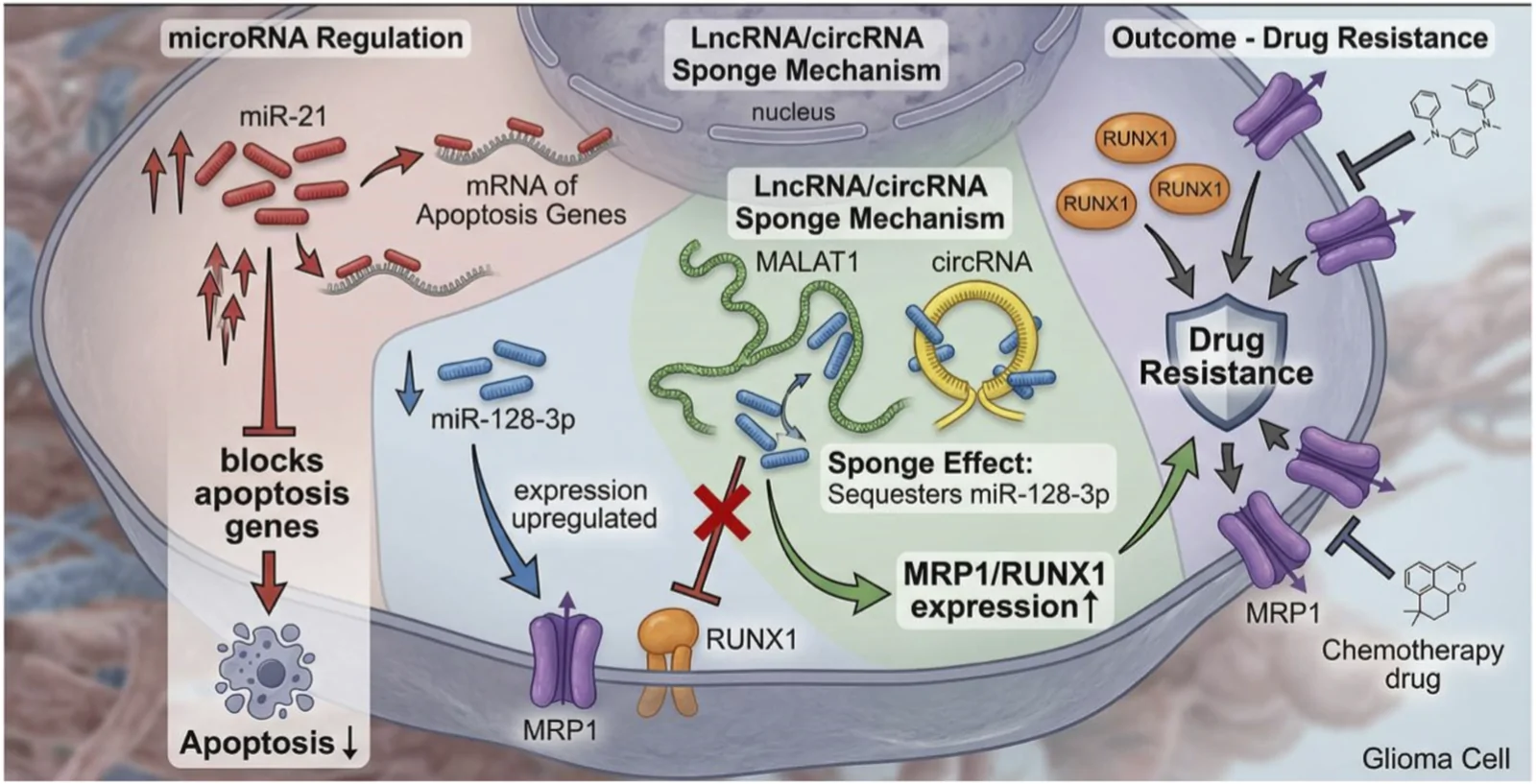
Regulatory network of non-coding RNA-mediated glioma chemoresistance: miR-21 inhibits apoptosis, while lncRNA/circRNA upregulates RUNX1/MRP1 through sponge adsorption of miR-128-3p to promote drug efflux.

The regulatory network structure involving ncRNA is complex, in which the competitive endogenous RNA (Cerna) interaction is one of the core regulatory modes - long non coding RNA (*lncRNA*) can be used as a molecular sponge of miRNA, and indirectly regulate the downstream gene expression and signal pathways that are important for glioma progression and drug resistance through competitive binding miRNA. For example, *lncRNA* *PVT1* promotes the drug resistance of glioma cells to temozolomide (TMZ) by regulating mir-365/elf4/sox2 axis, which highlights the therapeutic potential of destroying such Cerna axis (Gong et al., 2021). In addition, the positive feedback loop formed by *lncRNA* and key signaling pathways has also been confirmed to be closely related to TMZ resistance, such as the positive feedback regulation of *RMRP/ZNRF3*3 axis and wnt/β - catenin signaling pathway, suggesting that the drug resistance mechanism of glioma can be reversed by regulating ncRNA (Liu et al., 2021). In general, targeting these complex ncRNA mediated pathways is expected to re sensitize glioma cells to chemotherapy and radiotherapy, thereby improving the clinical prognosis of patients (Figure 5).

In conclusion, non coding RNA (ncRNA) has become a potential target molecule in the field of glioma treatment due to its key regulatory role in tumor biology and treatment resistance. Antisense oligonucleotides, miRNA mimics or inhibitors provide feasible targeting strategies for precisely regulating these ncRNAs. However, breaking through the delivery barrier of blood-brain barrier (BBB) and developing efficient and tumor specific delivery systems are still the key to the clinical success of ncRNA targeted therapy. In the future, the combination of ncRNA targeted therapy with traditional chemotherapy, radiotherapy and other treatment methods is expected to provide innovative and efficient treatment for glioma patients, and bring new hope for improving the prognosis of patients (Jin and Bai, 2025; Marangon and Lecca, 2023).

### 6.5 Non-coding RNA-mediated interactive regulatory network between Hippo and mitochondria

Mitochondrial dysfunction is by no means a passive metabolic phenomenon accompanying the process of tumor drug resistance; rather, it can actively initiate the cascade reaction of the Hippo pathway in its capacity as an upstream signal source. Functional experiments conducted by Kwon et al. have demonstrated that classical mitochondrial stress inducers such as CCCP, rotenone, and antimycin A can rapidly and thoroughly eliminate the phosphorylation modification of YAP/TAZ proteins, prompting their migration into the nucleus and subsequently triggering the specific transcription of a series of downstream target genes. Transcriptome data from RNA sequencing also corroborate this regulatory relationship: the expression and activation of the vast majority of mitochondrial stress-related transcripts within cells are highly reliant on the transcriptional regulatory activity of YAP/TAZ. We can discern a clear molecular signaling logic from this: the superoxide accumulated through mitochondrial stress directly oxidizes and modifies the RhoA protein, thereby activating the PP2A phosphatase and ultimately achieving the dephosphorylation regulation of YAP/TAZ (Cao et al., 2024).

This exclusive signaling axis, composed of “mitochondrial ROS → RhoA oxidation → PP2A activation → YAP/TAZ dephosphorylation → nuclear translocation,” fills a critical gap in the understanding of the physiological upstream activation mechanism of the Hippo pathway. It is perhaps this activation mode mediated by mitochondrial dysfunction that enables the Hippo pathway to persistently exert a pro-cancer effect and participate in shaping the malignant phenotype of gliomas.

The activation of the Hippo pathway by mitochondrial stress is not a unidirectional signaling process. Can the activated YAP/TAZ, in turn, regulate the physiological state of mitochondria and form a self-sustaining regulatory loop? Existing research findings provide an affirmative answer. Functional verification experiments have shown that the abnormal overexpression of YAP1 can significantly enhance overall mitochondrial function and cellular energy metabolism levels, with the most direct manifestations being an increase in mitochondrial membrane potential and a substantial rise in intracellular ATP synthesis (Bai et al., 2024). Conversely, specifically silencing the YAP1 gene can completely reverse these metabolic benefits and restore mitochondrial function to a suppressed state. From a molecular perspective, the Hippo-YAP signaling pathway can further initiate the transcriptional activation of PPA2 by upregulating the expression of the copper chaperone protein ATOX1, effectively eliminating excessive mitochondrial superoxide within cells, stabilizing mitochondrial membrane potential, ensuring normal ATP synthesis, and thereby repairing dysfunctional mitochondria (Yu et al., 2025). Additionally, the YAP protein possesses the ability to bidirectionally fine-tune mitochondrial metabolism: it can positively activate transcription factors related to mitochondrial biogenesis, such as Nrf1, RXRα, and POLG, while moderately inhibiting the abnormal expression of HIF-1α.

In fact, YAP/TAZ plays a dual role in this regulatory system, serving both as a molecular sensor for detecting mitochondrial dysfunction and as a core regulator for precisely correcting mitochondrial metabolic status. These two organelles and signaling pathways form a tightly coupled bidirectional regulatory relationship.

Hypoxia represents the most typical and central pathological feature of the glioma microenvironment. How do tumor cells convert hypoxia microenvironment signals into adaptive changes in internal metabolism and signaling pathways? NcRNA plays a pivotal intermediary role in this signal transduction process, establishing a regulatory pathway that connects hypoxia signals to mitochondrial metabolism and the Hippo pathway. *In vivo* and *in vitro* experiments on glioblastoma have demonstrated that hypoxia stress significantly induces the upregulation of miR-210 expression (Agrawal et al., 2014). The enriched miR-210 can directly target the mitochondrial functional enzyme ALDH5A1, effectively downregulating the expression level of this gene. Inhibition of ALDH5A1 expression directly reduces the oxygen consumption rate of tumor cells, increases the degree of extracellular acidification, and helps glioma cells maintain a high-glycolysis metabolic profile (Mondal et al., 2024). Conversely, overexpression of ALDH5A1 can effectively reverse this metabolic remodeling process.

Simultaneously, miR-210 can also target and inhibit the ISCU1/2 gene, disrupting the electron transport chain activity of mitochondrial complex I, further blocking oxidative phosphorylation, and promoting a shift in tumor cell metabolism towards glycolysis (Ho et al., 2022). In most cases, this cascade pathway of “hypoxia → miR-210 → mitochondrial metabolic remodeling” serves as an important metabolic foundation for glioma cells to adapt to hypoxic microenvironments and develop chemotherapy resistance.

The regulatory role of ncRNA is not confined to mitochondrial metabolism but also extends to directly targeting the core functional components of the Hippo pathway. miR-650 can facilitate the invasion and migration of glioma cells by inhibiting LATS2 expression, while miR-103a weakens the stability of the LATS2 protein by targeting the deubiquitinase OTUB1 (Ye et al., 2022). It is important to note that the inactivation of LATS1/2 kinase is the core prerequisite for the abnormal and sustained activation of YAP/TAZ.

Long non-coding RNAs and circular RNAs are also deeply involved in this regulatory network. For instance, MKLN1-AS can adsorb miR-126-5p through the endogenous competitive RNA mechanism, relieving its inhibitory effect on TEAD1 and ultimately activating the TEAD1-mediated Hippo pathway transcription program (Chen S. et al., 2025). Thus, the molecular chain of “ncRNA regulation → Hippo kinase inactivation → YAP/TAZ abnormal activation” successfully couples the ncRNA regulatory network with the classical Hippo signaling pathway.

By analyzing the aforementioned hierarchical regulatory mechanism, we can delineate a complete closed regulatory loop: the hypoxic microenvironment triggers the abnormal upregulation of various ncRNAs, including miR-210, which drives mitochondrial metabolic reprogramming characterized by the downregulation of ALDH5A1 expression and an elevation in cellular glycolysis levels. Mitochondria with metabolic disorders continuously accumulate ROS, inducing oxidative modification of the RhoA protein, activating PP2A phosphatase, and mediating the dephosphorylation of YAP/TAZ. Dephosphorylated YAP/TAZ translocates into the nucleus, initiating transcriptional reprogramming of downstream target genes, many of which are involved in mitochondrial function regulation, thereby reversely correcting and reshaping the mitochondrial physiological state (Nafe and Hattingen, 2023).

This integrated framework completely disrupts the notion that the three regulatory modules operate independently, unifying the Hippo signaling pathway, ncRNA regulatory network, and mitochondrial dysfunction into a cohesive glioma drug resistance-driving system. The core features of this network are multiple positive feedback loops and cross-amplification effects: activated YAP/TAZ can further upregulate the expression of hypoxia-inducible factors, indirectly reinforcing the abnormal expression of ncRNAs; the continuous accumulation of mitochondrial ROS sustains the activation state of YAP/TAZ, enabling the entire regulatory loop to self-reinforce and maintain stable operation, ultimately firmly preserving the chemotherapy resistance phenotype of glioma (Nafe and Hattingen, 2023).

Given the self-compensatory and amplification characteristics of this closed-loop network, it is evident that treatment strategies targeting a single network node have significant limitations. Whether inhibiting YAP/TAZ activity alone or blocking miR-210 function independently, tumor cells can compensate for pathway dysfunction through the loop’s compensatory regulatory mechanisms, ultimately leading to a substantial decline in therapeutic efficacy.

To effectively overcome the drug resistance barrier in glioma, should we shift towards a multi-node joint intervention approach? Based on the regulatory mechanism elucidated in this study, the answer is affirmative. Theoretically, a multi-dimensional joint intervention strategy based on the aforementioned closed-loop regulatory network may hold greater clinical potential: combining verteporfin to block YAP/TAZ transcriptional activity, employing antisense oligonucleotides (ASOs) to silence hypoxia-induced miR-210 and other key ncRNAs, and correcting mitochondrial metabolic shifts with dichloroacetic acid (DCA). This “transcription-signal-metabolism” triple-blocking approach is expected to simultaneously disrupt the tumor microenvironment adaptation mechanism at multiple key nodes, thereby more effectively breaking the drug resistance homeostasis of glioma. This mechanistic understanding provides reliable theoretical support for the design of combined glioma therapies. Meanwhile, there remains ample scope for further research in this field. Subsequent studies could analyze the quantitative dynamic characteristics of network nodes and elucidate the dynamic evolution patterns of the feedback loop over time, providing more detailed experimental evidence for optimizing the timing and schemes of individualized precision treatment for glioma.

## 7 Clinical mechanisms and translational challenges of drug resistance in glioma treatment

### 7.1 Clinical mechanisms and translational challenges associated with temozolomide resistance

Temozolomide (TMZ), a classic alkylating chemotherapy agent, exerts cytotoxic effects on tumor cells by modifying the O6 position of DNA guanine and generating methylated adducts. O6-methylguanine-DNA methyltransferase (MGMT) can directly eliminate these aberrant methylation modifications, repair damaged tumor DNA, and thereby diminish the antitumor activity of TMZ. Among all identified molecules predictive of TMZ efficacy, MGMT promoter methylation stands as the sole core biomarker validated through large-scale clinical cohorts and incorporated into the authoritative glioma diagnosis and treatment guidelines issued by both the NCCN and EANO. Clinical follow-up data confirm that glioblastoma patients with MGMT promoter methylation derive significant survival benefits from concurrent TMZ chemoradiotherapy, with median survival extended to 21.7 months; conversely, the clinical benefits of this regimen for non-methylated patients are negligible (Yan et al., 2026; Weller et al., 2021).

In reality, the clinical utility of MGMT promoter methylation is far less definitive than outlined in the guidelines. Clinical epidemiological data reveal that only 40%–50% of glioblastoma (GBM) cases exhibit this methylation modification, implying that over half of the patients inherently possess primary resistance to TMZ and thus cannot benefit from standard chemotherapy. Furthermore, the lack of uniformity in detection techniques further hampers the precise application of this biomarker. Different clinical laboratories employ mainstream detection methods such as methylation-specific PCR and pyrosequencing, with notable variations in equipment parameters and critical threshold settings, often leading to discrepancies in stratification results for the same patient. To mitigate such detection biases, the updated EANO guidelines have introduced a “gray zone” determination interval with a methylation ratio of 5%–12% to refine clinical decision-making. However, the generalizability and clinical stability of this optimized approach still require further validation through multicenter prospective trials (Weller et al., 2021).

MGMT promoter methylation status effectively predicts initial treatment response but fails to elucidate the mechanisms underlying drug resistance during later stages of tumor treatment. Many patients with typical MGMT methylation characteristics still experience tumor recurrence and acquired resistance following standard TMZ therapy, indicating that MGMT-independent regulatory pathways are also deeply implicated in TMZ resistance. Recent genome-wide CRISPR screening studies have systematically delineated the core framework of these bypass mechanisms: the DNA damage repair network (ATRX-PARP1 signaling axis, RAD51AP1-mediated homologous recombination repair), tumor cell stress adaptation mechanisms, glioma stem cell stemness maintenance, and intratumoral heterogeneity collectively constitute the core driving system of TMZ resistance (Macle et al., 2019).

Clinical real-world data can more vividly illustrate this treatment dilemma. Why do over 90% of GBM patients initially respond well to TMZ treatment, yet ultimately succumb to early recurrence? The answer likely lies in the persistently activated non-specific drug resistance pathways. The prognosis for patients after recurrence is extremely poor, with a median survival time of less than 8 months, representing a core bottleneck in current GBM clinical treatment (Smerdi et al., 2024).

Drug efflux transport was an early focus of academic research on drug resistance mechanisms. ABCB1 (P-glycoprotein), as the core functional transporter of the blood-brain barrier, has long been hypothesized to actively efflux intracranial TMZ, thereby reducing drug accumulation within tumor tissue and weakening the efficacy of chemotherapy. However, continuously updated clinical cohort data have overturned this classic preclinical hypothesis. A systematic review involving over 400 GBM patients confirmed that high-frequency polymorphisms in the ABCB1 gene, such as C1236T, 2677G > T/A, and 3435C > T, do not significantly impact treatment response or survival prognosis. Under the combined influence of strong prognostic markers like MGMT methylation status and IDH gene mutations, the drug resistance mediated by the ABCB1 transporter alone exerts minimal clinical impact (De Luca et al., 2026). This finding underscores that the drug efflux resistance mechanisms validated by most preclinical models exhibit notable laboratory-based biases, and their significance in real-world clinical settings may have been overestimated in previous studies. Researchers should re-evaluate the clinical relevance of these traditional drug resistance pathways by considering the spatial metabolic characteristics of intracranial drugs and the stratification of patients based on molecular typing.

The metabolic adaptive remodeling of tumor cells represents a novel breakthrough in elucidating the mechanisms of TMZ resistance in recent years. Drug-resistant GBM cells actively reconstruct their metabolic patterns, characterized by abnormal activation of the glycolytic pathway, sustained hyperactivity of the antioxidant system, and overall remodeling of the lipid metabolism profile (Yan et al., 2026). Specifically, the upregulated expression of key glycolytic genes such as GLUT3, HK2, PKM2, and LDHA significantly enhances cellular glycolytic flux, providing ample energy and material substrates for the proliferation and repair of drug-resistant tumor cells.

Among various metabolic regulatory molecules, the lactic acid accumulation mediated by LDHA holds unique significance in drug resistance regulation. Lactic acid secreted by tumor cells induces lactylation modification of histone H3K9. This epigenetic modification specifically inhibits the transcription and expression of MLH1, a core molecule in the mismatch repair (MMR) pathway, thereby directly blocking the DNA damage response induced by TMZ. Notably, this metabolic-epigenetic cross-regulatory pathway operates independently of the methylation status of the MGMT gene. Could this explain the clinical conundrum of drug resistance observed in some patients with MGMT methylation? Existing research evidence supports this hypothesis and fills the gap in drug resistance mechanisms not covered by the classical MGMT pathway (Yue et al., 2024).

Clinical translation efforts based on this mechanism have been initiated. The glycolytic inhibitor dichloroacetic acid (DCA) has completed a phase I clinical trial for recurrent high-grade gliomas. The trial results confirm that oral administration of DCA is safe and feasible, with good overall patient tolerance. However, this study did not demonstrate ideal antitumor benefits, indicating that the therapeutic efficacy of single metabolic intervention remains limited (NCT00703859) (Lesueur et al., 2019). This underscores that drug resistance mediated by tumor metabolic reprogramming is a complex network-regulated process, and single-target interventions are unlikely to fully reverse the clinical drug resistance phenotype.

Drawing on years of accumulated research on mechanisms, methylation detection of the MGMT promoter has been officially included in multiple authoritative diagnosis and treatment guidelines from NCCN, ASCO-SNO, and EANO. It has become the cornerstone for individualized hierarchical treatment of GBM patients, directly guiding the formulation of clinical differentiated medication regimens (Weller et al., 2021; Gajjar et al., 2025). Currently, the prevailing clinical hierarchical treatment logic can be categorized into three types: for newly treated patients aged 70 or younger with good physical function, clinical treatment does not necessitate consideration of MGMT modification status, and a standard radiotherapy regimen combined with synchronous and adjuvant TMZ chemotherapy is uniformly adopted; for elderly patients over 70 with acceptable functional status, only those with MGMT methylation are suitable for combined radiotherapy and chemotherapy, whereas those without methylation are better suited for radiotherapy alone; for elderly and frail patients with poor physical function, treatment can be simplified based on MGMT status, with methylated patients receiving TMZ chemotherapy alone and non-methylated patients prioritizing radiotherapy alone or optimal supportive care.

Focusing on the classic drug resistance pathway mediated by MGMT, the academic community has conducted multiple rounds of clinical trials involving pharmacological interventions, yet the overall translational outcomes have consistently fallen short of expectations. O6-benzylguanine (O6-BG) can effectively restore tumor cell sensitivity to TMZ by depleting intracellular functional MGMT protein. In a phase II clinical trial combined with Gliadel sustained-release wafers, patients achieved an 82% 6-month overall survival rate and an extended median survival of 50.3 weeks. However, severe dose-limiting bone marrow suppression and a 13.4% incidence of intracranial infections significantly hinder the widespread clinical adoption of this regimen (Quinn et al., 2009).

PARP inhibitors represent another class of promising sensitizing agents that amplify the cytotoxic effects of chemotherapy by blocking the base excision repair pathway, thereby exacerbating the accumulation of TMZ-induced tumor DNA damage. A clinical trial of pamiparib combined with low-dose TMZ demonstrated a median overall survival of 12.8 months in newly diagnosed MGMT-unmethylated GBM patients, although treatment safety concerns persisted, with a 55% incidence of grade ≥3 adverse events (Piotrowski et al., 2025). The phase I/II clinical trial of olaparib combined with concurrent TMZ chemoradiotherapy is ongoing, and complete survival and safety data (NCT03212742) have yet to be published (Lesueur et al., 2019).

From a series of clinical explorations marked by mixed outcomes, it is evident that the tumor heterogeneity of GBM far exceeds expectations, rendering targeted intervention strategies against a single drug-resistance pathway ineffective in achieving long-term and stable anti-tumor effects. Current mechanistic research findings are reshaping the clinical treatment landscape of GBM across three dimensions.

Firstly, the precise stratified treatment system continues to be refined. The foundational stratification model, based on MGMT methylation and IDH mutation status, has been integrated into standardized clinical diagnosis and treatment protocols. To further enhance stratification accuracy, it may be necessary to incorporate novel drug-resistance markers such as RAD51AP1 and ATRX, thereby establishing a multi-dimensional molecular typing system.

Secondly, combination targeted therapies have emerged as the mainstream of research and development. A wealth of preclinical data confirms that the combined intervention of metabolic regulators with traditional radiotherapy and chemotherapy can block tumor drug resistance through multiple pathways. However, there remains a dearth of large-sample, multicenter Phase III clinical trials that can directly validate the clinical survival benefits of this combined strategy (Yan et al., 2026; Yue et al., 2024).

Thirdly, dynamic drug-resistance monitoring has become a new research frontier. Circulating LDHA-positive exosomes and other novel drug-resistance markers are closely associated with adverse treatment responses and shortened survival periods in patients, endowing liquid biopsy technology with potential application value for real-time monitoring of tumor drug-resistance progression. Compared to traditional postoperative tissue biopsies, noninvasive liquid biopsies enable dynamic follow-up throughout the treatment process. Will they become the core means for early warning of GBM drug resistance and adjustment of treatment plans in the future? This direction warrants continuous exploration and validation.

### 7.2 Clinical trial progress and translational challenges in targeting drug resistance mechanisms

#### 7.2.1 MDM2-p53 pathway inhibitor

Navtemadlin—p53 serves as the core tumor suppressor protein essential for maintaining genome stability, with MDM2 acting as its key negative regulator. Given that approximately 60% of glioblastomas (GBMs) retain wild-type p53, restoring p53 function through MDM2 inhibitors is theoretically viable. A phase I clinical trial (NCT03107780) assessed the pharmacokinetics and safety of Navtemadlin in both relapsed and newly treated GBM patients. Part 1 confirmed that Navtemadlin could penetrate the blood-brain barrier and achieve effective therapeutic concentrations (≥25 nmol/L) in tumor tissues; Part 2 explored the dosing of Navtemadlin combined with radiotherapy in patients with newly diagnosed, MGMT promoter non-methylated, and p53 wild-type GBM (Sandbhor et al., 2023). The results indicated that while the drug effectively activated the intracranial p53 pathway, its efficacy as a monotherapy or in combination with radiotherapy was limited, suggesting the need to explore combination therapy with temozolomide (TMZ) and other DNA-damaging agents to overcome drug resistance.

#### 7.2.2 Base excision repair (BER) inhibitor

TRC102 Combined with TMZ (BERT Trial) - The BER pathway represents one of the non-MGMT-dependent mechanisms mediating TMZ resistance. The multicenter phase II BERT trial (NCT02395692) enrolled 19 GBM patients experiencing their first relapse after initial bevacizumab treatment and evaluated the efficacy of TRC102 (methoxyamine) combined with TMZ. The results revealed no objective responses, with a median overall survival (OS) of 11.1 months and a median progression-free survival (PFS) of 1.9 months. Notably, two patients exhibited long-term survival benefits (PFS ≥17 months, OS > 32 months) (Rehman et al., 2022). Transcriptomic analysis demonstrated significant enrichment of DNA damage response (DDR) and chromosomal instability (CIN) features in the baseline tumors of these “long-term survivors.” The trial indicated that the TRC102 + TMZ regimen was safe and feasible, but its efficacy might be confined to specific molecular subtypes with high DDR pathway expression.

#### 7.2.3 Hedgehog pathway inhibitor

Glasdegib Combined with the Stupp Regimen (GEINO 1602 Trial)—The Hedgehog pathway plays a pivotal role in maintaining the stemness of glioma stem cells (GSCs) and mediating resistance to chemoradiotherapy. The phase Ib/II GEINO 1602 trial (NCT03466450) evaluated the efficacy of the SMO inhibitor Glasdegib combined with the standard Stupp regimen in newly diagnosed GBM patients. Phase II results showed a 15-month OS rate of 52.1%, failing to surpass the predefined efficacy threshold (60%); however, the 24-month OS rate reached 29.2%, with a median OS of 15.3 months. Subgroup analysis revealed a median OS of 22.9 months for patients with MGMT promoter methylation and 14.1 months for those without methylation (Liu et al., 2024). Although the primary endpoint was not met, the long-term survival data suggested that Hedgehog pathway inhibition might benefit specific patient subgroups, and ongoing translational research is focused on identifying biomarkers for responsive populations.

LAG-3+PD-1 Dual Immune Blockade (ABTC 1501 Trial)—Given the limitations of PD-1 monoclonal antibody monotherapy in recurrent glioblastoma multiforme (GBM), LAG-3 has garnered attention as a pivotal compensatory immune checkpoint. The Phase I ABTC 1501 trial (NCT02658981) assessed the safety and biological activity of the anti-LAG-3 antibody relatlimab in combination with nivolumab in recurrent GBM. The results indicated that the combination therapy was safe and manageable, with neoadjuvant administration significantly enhancing the infiltration of CD8^+^ T cells within the tumor. Clinical data revealed that the median overall survival (OS) in the combination group reached 16.7 months, with a 12-month OS rate of 52.2%, outperforming historical controls (Zhang et al., 2022). Based on these findings, the Phase II GIANT trial (NCT06816927) evaluating nivolumab ± relatlimab in the perioperative treatment of newly diagnosed IDH wild-type GBM has been initiated.

#### 7.2.4 MET inhibitor

Vebreltinib for ZM Fusion Gene-Positive Gliomas (FUGEN Study) - The PTPRZ1-MET (ZM) fusion gene represents a significant driver mutation in high-grade gliomas. The multicenter, randomized controlled Phase III FUGEN study (NCT02829998) enrolled 84 patients with ZM fusion-positive high-grade gliomas who had failed prior treatments. The results demonstrated that the median OS in the vebreltinib group was significantly superior to that in the chemotherapy control group (6.3 months vs. 3.4 months; HR = 0.52, P = 0.007). In the subgroup with a baseline tumor diameter ≤3.0 cm, vebreltinib exhibited an even more pronounced survival benefit (median OS 32.5 months vs. 4.2 months) (D’amico and De amicis, 2022). Based on the groundbreaking data from this study, vebreltinib received approval from the China National Medical Products Administration (NMPA) in December 2024 for the treatment of PTPRZ1-MET fusion-positive gliomas that have failed prior treatments, marking it as the world’s first approved targeted therapy for glioma indications (Table 1).

**TABLE 1:** A summary of clinical trials and translational research on drug resistance mechansms in glioma treatment.

Research title/drug	Target/action mechanism	Experimental phase	Target audience	Primary outcomes (OS/PFS/others)
Navtemadlin	Inhibitors of the MDM2-p53 pathway; restore p53 functionality	Phase I (NCT03107780)	Recurrent and newly treated GBM; part 2 targets patients with newly diagnosed GBM, non-methylated MGMT, and wild-type p53	Drugs are capable of penetrating the blood-brain barrier; however, the efficacy of single-agent or combined radiotherapy is limited.
BERT trial (TRC102 + TMZ)	Base excision repair (BER) inhibitors; blocking the non-MGMT-dependent drug resistance pathway	Multicenter phase II trial (NCT02395692)	Nineteen GBM patients experiencing their first relapse and receiving initial treatment with bevacizumab	Median overall survival (OS): 11.1 months; median progression-free survival (PFS): 1.9 months; No objective remission observed; two patients achieved long-term survival (PFS ≥ 17 months).
GEINO 1602 trial (Glasdegib + Stupp regimen)	Inhibitors of the hedgehog pathway (SMO); suppression of stemness and drug resistance in glioma stem cells	Phase Ib/II (NCT03466450)	Patients newly diagnosed with GBM	15-month overall survival (OS) rate: 52.1% (failing to reach the preset threshold of 60%); 24-month OS rate: 29.2%; median OS: 15.3 months. Subgroup analysis: The median OS was 22.9 months in the MGMT-methylated group and 14.1 months in the non-methylated group.
ABTC 1501 trial (relatlimab + nivolumab)	Dual immune blockade targeting LAG-3 and PD-1; enhanced infiltration of T cells	Phase I (NCT02658981)	Patients with recurrent glioblastoma multiforme (GBM)	Median overall survival (OS): 16.7 months; the 12-month OS rate stood at 52.2% (superior to the historical control); neoadjuvant therapy enhances intratumoral infiltration of CD8^+^ T cells.
FUGEN study (Beretinib/Vebreltinib)	MET inhibitors; mutations driven by the PTPRZ1-MET (ZM) fusion gene	A multicenter, randomized, controlled phase III trial (NCT02829998)	Eighty-four patients with ZM fusion-positive high-grade gliomas who had failed previous treatments	The median overall survival (OS) in the beretinib group was significantly longer than that in the chemotherapy group (6.3 vs. 3.4 months; HR = 0.52, P = 0.007). In the subgroup with baseline tumors ≤3.0 cm, the median OS reached 32.5 months.
O6-benzylguanine (O6-BG)	MGMT protein depleting agent; restore tumor cell sensitivity to TMZ	Phase II clinical trial exploration	GBM patients with MGMT-mediated drug resistance (treated in combination with Gliadel sustained-release wafers)	The 6-month overall survival rate stood at 82%; the median survival time was 50.3 weeks
Pamiparib combined with low-dose TMZ	PARP inhibitors; they block base excision repair and exacerbate DNA damage.	Clinical studies	Newly diagnosed GBM patients with unmethylated MGMT status	Median overall survival (OS): 12.8 months
Dichloroacetic acid (DCA)	Glycolysis inhibitors; intervention in drug resistance mediated by metabolic reprogramming	Phase I (NCT00703859)	Patients suffering from recurrent high-grade gliomas	Oral administration is safe, feasible, and well-tolerated; however, it did not demonstrate ideal anti-tumor benefits.

OS, overall survival; PFS, progression-free survival; GBM, glioblastoma; TMZ, temozolomide; MGMT, O6-Methylguanine-DNA, methyltransferase; DDR, DNA, damage response; NMPA, China National Medical Products Administration. Data Description: The unit for survival periods (OS, PFS) in the table is “months.” Some trials with incomplete data published (such as olaparib combined with TMZ) are not included in this table.

### 7.3 Challenges and insights from transformation

The precise selection of biomarkers directly determines the effectiveness and ultimate outcomes of glioma clinical trials. Sample analysis from the BERT trial, focusing on cancer patients with long-term survival, revealed that a highly expressed DDR pathway can form a unique molecular signature. Could this phenomenon offer precise subtype screening criteria for optimizing subsequent combination therapies? It may serve as a pivotal starting point for subsequent clinical validation. The overall data from the GEINO 1602 trial did not surpass the threshold for clinical ineffectiveness, failing to demonstrate the trial’s benefit to the entire patient population. However, subgroup analysis yielded contrasting results. In this trial, patients with MGMT methylation experienced an extended overall survival of 22.9 months, indicating that hierarchical analysis based on molecular characteristics can uncover treatment benefits concealed within the overall trial data (Liu et al., 2024).

The combined use of various therapies has emerged as the mainstream trend in glioma clinical treatment research and development. Yet, despite the efficacy gains, managing treatment toxicity remains an unavoidable core challenge. When used alone, Navtemadlin provides relatively modest clinical anti-tumor benefits, making it difficult to achieve the desired therapeutic effect independently. In fact, when combined with TMZ, the drug effectively activates tumor cell apoptosis pathways, significantly enhancing the anti-tumor effect. The dual immune blockade strategy involving LAG-3 and PD-1 also demonstrated superior survival benefits compared to single-agent immunotherapy. However, the safety concerns associated with combination therapy are prominent, with 26% of patients in the combination regimen experiencing grade 3–4 severe adverse events. This necessitates that clinical research and development meticulously weigh the balance between therapeutic benefits and medication safety while pursuing curative breakthroughs (Zhang et al., 2022).

The high degree of tumor heterogeneity has consistently been a core barrier to achieving breakthroughs in glioma treatment. In most clinical explorations, single-target drugs struggle to cover all tumor cell populations. Classic single-target drugs such as Hedgehog inhibitors and BER inhibitors exhibit limited efficacy only in a small subset of patients and fail to effectively improve the overall prognosis of glioblastoma patients. The clinical implementation of Beretinib has validated the feasibility of precise targeted therapy for rare driver genes, offering a novel approach to glioma precision treatment (D’amico and De amicis, 2022). However, this drug’s applicability covers only 2%–5% of patients. Does such a narrow scope of application imply that a single genetic classification can no longer meet current clinical treatment needs? This also underscores the necessity for the industry to establish a multi-dimensional and comprehensive molecular classification system to tailor individualized treatment plans for different patients.

Targeted drug delivery strategies with optimization are progressively unlocking the clinical potential of glioma therapies. The exclusive structural design enabling blood-brain barrier penetration is a pivotal factor that allows beretinib to surpass the limitations of conventional drug treatments and deliver clinical benefits. In the drug development for most central nervous system tumors, mere target precision is no longer sufficient to substantiate the clinical value of drugs. Achieving simultaneous optimization of drug chemical structures and precise target engagement may serve as an exemplary model for future research and development of novel glioma drugs.

## 8 Strategies for overcoming drug resistance of glioma based on nanotechnology

### 8.1 Nano carrier drug delivery system

Nanocarrier drug delivery system has become a key strategy to overcome the major challenge of blood-brain barrier (BBB) in glioma treatment. As a highly selective physiological barrier, the blood-brain barrier will strictly restrict the passage of most chemotherapy drugs, resulting in insufficient effective concentration of drugs at the glioma site, which will significantly reduce the therapeutic effect. At present, a variety of nano carriers have been successfully developed, including nanoparticles, liposomes, dendrimers and polymer systems. These carriers can effectively promote the drug to cross the blood-brain barrier, which can not only significantly improve the drug bioavailability at the tumor site, but also minimize the systemic toxicity of drugs, providing a new breakthrough for the treatment of glioma.

Among various nano carriers, liposomes and polymer nanoparticles show significant potential in improving the permeability of blood-brain barrier. With its good biocompatibility and the unique advantages of simultaneously containing hydrophilic and hydrophobic drugs, liposomes can further enhance tumor targeting specificity and *in vivo* circulation time through surface modification. For example, lipid based nanocarriers have been successfully developed for drug delivery across the blood-brain barrier (Qiao et al., 2022). Similarly, polymer nanoparticles that respond to redox conditions can achieve controlled drug release and avoid premature drug release at non target sites, thus significantly improving the therapeutic effect (Ismail et al., 2024).

Dendrimers provide multiple binding sites for drug molecules and target molecules by virtue of their highly branched and surface modifiable structural characteristics. Their nano size and surface functionalization make them able to penetrate the blood-brain barrier efficiently and deliver therapeutic agents directly to glioma cells. It has been reported that the carrier based on dendrimer can be coupled with peptide or transferrin for image-guided treatment of glioma and realize the synergy of diagnosis and treatment (Wen et al., 2024). In addition, biomimetic nanoparticles coated with natural cell membrane or simulating endogenous vesicles (such as exosomes) have also been proved to significantly enhance the targeting of glioma cells and effectively improve the efficiency of drug delivery (Rana et al., 2025; Zhang G. et al., 2023).

Lipid nanoparticles (LNPS) have attracted much attention because of their advantages of encapsulating chemotherapeutic drugs and genetic materials at the same time, which can realize the combined application of chemotherapy, immunotherapy, photothermal therapy and other treatment modes. Among them, the intelligent lipid nanoparticles designed to overcome the chemoresistance and radiation resistance of glioma have shown the potential to improve the therapeutic effect of glioma in relevant studies (Bera et al., 2025); In addition, solid lipid nanoparticles developed for glioma treatment not only show good tumor accumulation characteristics, but also have low systemic toxicity, which further improves the safety of treatment (Battaglia et al., 2025).

The application of ligand modification strategy has further improved the targeting specificity of nanocarriers for glioma cells. Commonly used modification ligands include lactoferrin, transferrin, folic acid and heat shock protein 70 (HSP70). These targeted ligands can effectively break through the blood-brain barrier by promoting receptor-mediated endocytosis, and preferentially enrich in glioma tissues. For example, lactoferrin modified periodic mesoporous silicone nanoparticles can significantly enhance the uptake of glioma cells, thereby improving the therapeutic effect of doxorubicin (Dong et al., 2023); Similarly, HSP70 targeted nanoparticles combined with immune checkpoint inhibitors have shown good synergistic effects in animal models, which can not only effectively induce glioma cell apoptosis, but also prolong the survival time of model animals (Xie et al., 2023).

In addition to the optimization of the carrier itself, the innovative route of administration also provides a new idea for breaking through the blood-brain barrier. Intranasal administration bypasses the blood-brain barrier in a non-invasive way by using the olfactory nerve and trigeminal nerve pathway, avoiding the limitations of intravenous administration. Nano carriers specially designed for nasal administration, including metal nanoparticles and polymer nanoparticles, have shown enhanced brain targeting and low toxicity (Deshmukh et al., 2025), providing a promising alternative for the direct delivery of chemotherapy drugs to the brain.

In addition, researchers have also developed a hybrid nano platform with multiple functions, which integrates the functions of drug loading, imaging and stimulus response release, so as to realize the integration of diagnosis and treatment and further optimize the treatment effect. For example, biomimetic metal organic frameworks (MOFs) coated with brain targeting peptides can effectively penetrate the blood-brain barrier, accurately deliver chemotherapy drugs such as docetaxel, and significantly inhibit the growth of glioma in preclinical models (Wu et al., 2023); Similarly, the nanofiber system encapsulated with temozolomide shows sustained drug release and enhanced cellular uptake, which provides a potential improvement for the treatment of glioblastoma multiforme (Shetty and Yadav, 2024). At the same time, natural polysaccharide nano carriers (such as carriers derived from hyaluronic acid, chitosan and alginate), with their good biocompatibility and biodegradability, provide a safe and reliable platform for drug delivery. These carriers can achieve targeted drug delivery and controlled release, regulate tumor microenvironment and enhance immune regulation (Li C. et al., 2025), and their colloidal properties (including particle size and surface charge) have a key impact on the penetration of blood-brain barrier and the stability of nano carriers.

In conclusion, the nanocarrier drug delivery system provides a multi-dimensional solution for glioma treatment by enhancing drug penetration through the blood-brain barrier, achieving tumor specific targeting and controlling drug release. The integrated application of targeted ligands, stimulus response materials and biomimetic coatings has significantly improved the therapeutic effect and safety. With the continuous progress of nanotechnology, combined with the in-depth understanding of the biological characteristics of glioma and the dynamic mechanism of blood-brain barrier, it is expected to develop more effective and personalized treatment strategies for glioma patients in the future. In order to fully release the therapeutic potential of these nano carriers, it is urgent to further explore their clinical application value (Gao et al., 2021).

### 8.2 Combined administration and drug resistance transporter inhibition

Overcoming drug resistance in glioma treatment faces many challenges. The physiological barrier of blood-brain barrier and the abnormal activity of multidrug resistance (MDR) transporters further exacerbate this dilemma. Among them, ATP binding cassette transporters (such as P-glycoprotein, breast cancer resistance protein and other efflux pumps) are the most critical. These transporters can actively discharge chemotherapy drugs from glioma cells and brain microenvironment, directly limit the accumulation of drugs at the target site and weaken the therapeutic effect. To solve this problem, the research focus in recent years has focused on the combination of nano carrier mediated drug delivery technology and pharmacological inhibition of MDR transporters, so as to enhance the ability of drug penetration and retention in glioma tissues and provide a new path for reversing drug resistance.

Various drug delivery systems based on nanoparticles, including liposomes, solid lipid nanoparticles, polymer micelles and biomimetic exosomes, have shown significant potential in breaking through the blood-brain barrier and achieving precise drug delivery by glioma cells. For example, researchers designed double receptor specific exosomes to achieve the co delivery of temozolomide (TMZ) and O6 benzylguanine (BG) through the modification of functional vascular peptide-2 and CD133 RNA aptamer, which not only significantly enhanced the drug uptake ability of glioma stem cells and improved the blood-brain barrier permeability, but also successfully overcome the TMZ resistance mediated by the activity of O6 alkylguanine DNA transaminase (AGT) (Liang et al., 2022). Other studies have shown that ultra small solid lipid nanoparticles loaded with paclitaxel, regofinil and cerium nanoparticles can effectively inhibit the proliferation and angiogenesis of glioma cells in animal models, and can achieve efficient accumulation at glioma sites (Battaglia et al., 2025).

One of the core strategies to enhance the accumulation of drugs in tumor sites is to encapsulate MDR transporter inhibitors and chemotherapy drugs in nano carriers to achieve collaborative delivery. When PEGylated liposomes were co loaded with TMZ and O6 benzylguanine, the uptake rate and cytotoxicity of U87 glioma cells were significantly improved compared with free drugs, and the drug resistance mechanism of tumor cells was successfully reversed (Hegde et al., 2024). In addition, researchers have developed functional liposomes modified by ligands such as btp-7 and Br2 for the combined delivery of photosensitizers and chemotherapy drugs. This strategy can not only enhance the penetration ability of blood-brain barrier, but also promote the accumulation of drugs in cells, and ultimately achieve a synergistic therapeutic effect (Jing et al., 2025). This combined drug delivery mode not only effectively improves the drug concentration in glioma cells, but also reduces the systemic toxicity of drugs through tumor targeted delivery and improves the treatment safety.

The inhibitory effect of efflux transporters can be further verified by probenecid derivatives. These drugs can target a variety of ABC transporters, which have been proved to increase the accumulation of chemotherapy drugs in glioma cells and induce tumor cell apoptosis (Huttunen et al., 2024). In addition to chemical inhibitors, resveratrol and other natural compounds also show good resistance reversal potential, which can regulate the expression of P-glycoprotein and inhibit the activity of PI3K/Akt pathway, thereby enhancing the sensitivity of glioblastoma cells to chemotherapy drugs (Zhang Y. et al., 2023). These results indicate that the combination of MDR transporter inhibition and nano carrier mediated drug delivery technology is an effective means to overcome drug efflux mediated glioma resistance.

The innovative nano platform also realizes the controllable drug release in the tumor microenvironment by integrating the stimulus response characteristics, and further improves the reversal effect of drug resistance. For example, near infrared (NIR) pH and photoresponsive nanomaterials are co assembled with chemotherapeutic drugs and photothermal agents. With the help of multimodal synergistic mechanisms such as glutathione depletion and photothermal therapy, they significantly enhance the cytotoxicity to glioma cells, effectively improve the drug retention capacity and overcome drug resistance (Cao et al., 2025). Similarly, macrophage membrane coated nanoparticles combined with low-frequency ultrasound irradiation can promote the opening of blood-brain barrier and targeted drug delivery, significantly increase the drug accumulation in glioma model and strengthen the tumor inhibitory effect (Lin et al., 2024).

In addition, the combined delivery of chemotherapeutic drugs and gene therapy agents using nano carriers has also become an important research direction to improve the therapeutic effect of drug-resistant glioma. Nanosheets modified by rabies virus glycoprotein peptide (RVG) were used to deliver temozolomide and mir-375 (a microRNA that can promote glioma cell apoptosis), which not only achieved better blood-brain barrier penetration and glioma targeting, but also produced significant synergistic anti-tumor effect (Yang T. et al., 2024). This multifunctional strategy fully proves that the combination of drug delivery and drug resistance pathway molecular inhibitors can effectively enhance the chemotherapy sensitivity of glioma cells.

It is noteworthy that the combination of nanocarriers and MDR transporter inhibitors can not only promote drug accumulation, but also regulate the tumor microenvironment and immune response, so as to achieve multi-dimensional reversal of drug resistance. For example, the study of targeted liposome co delivery of artesunate and temozolomide showed that the system effectively inhibited DNA repair pathway and enhanced blood-brain barrier penetration through low-density lipoprotein receptor-mediated transcellular transport. In the TMZ resistant glioma model, it significantly improved the tumor cell apoptosis rate and the survival rate of model animals (Ismail et al., 2022). These findings suggest that a reasonably designed co delivery system can simultaneously block multiple drug resistance mechanisms and improve the therapeutic effect.

Based on the existing research results, the treatment strategy of nano carrier combined with MDR transporter inhibitor is an effective way to overcome the chemotherapy resistance of glioma. This strategy can significantly improve the treatment effect by enhancing the accumulation of drugs in the tumor site, achieving accurate targeted delivery and regulating the drug resistance related pathways, which provides the possibility to improve the prognosis of patients. Future research should focus on optimizing such delivery systems and promoting their clinical transformation, including evaluating the safety of the system, clarifying the pharmacokinetic characteristics, and verifying the combined therapeutic effect in different glioma subtypes.

### 8.3 Combined application of nanotechnology and hyperthermia

Magnetic nanoparticle mediated magnetic hyperthermia (MHT) has become an effective strategy to enhance drug retention in glioma cells and overcome drug resistance. Iron oxide nanoparticles (fenps) prepared by green synthesis using plant extracts (such as Sophora flavescens) have excellent magnetic and biocompatibility, and are ideal candidates for magnetic hyperthermia (Kizilbey et al., 2024). When these nanoparticles are exposed to alternating magnetic fields, they can produce local thermal effects, which can raise the temperature of tumor tissue to the level of cytotoxicity. This thermal effect can not only directly induce the death of tumor cells, but also increase the permeability of cell membrane and promote the uptake and retention of chemotherapy drugs in glioma cells. The precise local thermal effect of magnetic nanoparticles in tumor tissue provides a new idea to deal with the treatment problems such as the strong invasion of glioma and the limitation of blood-brain barrier (Hu et al., 2023).

The synergistic combination of nanotechnology and hyperthermia is not limited to simple physical tumor ablation. Through engineering design, nanoparticles can realize the collaborative delivery of chemotherapeutic drugs and hyperthermia function, form a dual therapeutic impact, and further improve the reversal effect of drug resistance. For example, nano lipid carriers functionalized with targeted ligands (such as bevacizumab modified docetaxel nanoparticles) have been proved to be more cytotoxic to glioblastoma cells by improving drug delivery efficiency and prolonging drug release time (Di Filippo et al., 2022). When combined with magnetic hyperthermia, this kind of multifunctional nano platform can make tumor cells more sensitive to chemotherapy-induced apoptosis through local heating, so as to effectively break through the mechanism of drug resistance. At the same time, heat stress produced by hyperthermia can also destroy the cell homeostasis, degrade the related proteins involved in the drug resistance pathway, and further enhance the curative effect of chemotherapy.

In addition, hyperthermia induced by magnetic nanoparticles can also promote apoptosis of glioma cells through heat shock reaction and oxidative stress. The latest research shows that hyperthermia can downregulate the expression of HSP-70 and other heat shock proteins, which are the key factors for tumor cells to produce heat resistance and drug resistance. Nano platforms containing curcumin and other natural compounds have been proved to inhibit the expression of HSP-70, thereby improving the efficiency of photothermal conversion and singlet oxygen generation, and ultimately enhancing the apoptosis level of glioblastoma cells (Huo et al., 2025). This dual mechanism of thermal ablation combined with molecular sensitization provides a promising solution for overcoming the problem of multidrug resistance in glioma.

The combination of magnetic hyperthermia and nanotechnology also provides precise space-time regulation for glioma treatment. By modifying targeted molecules on the surface of nanoparticles, they can penetrate the blood-brain barrier and selectively enrich in glioma tissue. Local precise hyperthermia can be achieved when an external magnetic field is applied. This targeted therapy model can not only reduce the systemic toxicity of drugs and protect healthy brain tissue, but also successfully break through the main limitations of traditional therapy (Grzegorzewski et al., 2025). In addition, the heat generated by hyperthermia can also regulate the tumor microenvironment, improve the local oxygenation state and promote the infiltration of immune cells, which are helpful to further overcome the treatment resistance.

In general, the combination of magnetic nanoparticles mediated hyperthermia and nanotechnology based drug delivery constitutes a multi-level treatment strategy, with the core goal of enhancing intracellular drug retention and promoting glioma cell apoptosis. With the unique physical and chemical properties of nanoparticles, this method can achieve targeted and controllable hyperthermia intervention, and then destroy the drug resistance pathway and improve the treatment effect. The continuous development and clinical transformation of such nanoparticles have brought new hope for glioma treatment (Huo et al., 2025).

### 8.4 Application of nanotechnology in cancer stem cell targeted therapy

Glioma stem cells (GSCs) are the core functional subsets of glioma, especially glioblastoma multiforme, which play a key role in the process of tumor occurrence, treatment resistance and recurrence. Therefore, targeted clearance of GSCs is the core breakthrough to reduce the risk of glioma recurrence and improve the prognosis of patients. Traditional therapies are often difficult to eradicate such drug-resistant cells, and the development of nanotechnology provides an effective solution for the design of targeted drug delivery systems that specifically recognize and eliminate GSCs. Precise targeted treatment of glioma can be achieved by functional design of nanoparticles, encapsulation of chemotherapy drugs, Gene Silencers or signal pathway inhibitors (Sabu et al., 2023).

In the nano medical research of GSCs targeted therapy, the surface modification of nanoparticles by ligands or antibodies is one of the key advances. These modified molecules can specifically recognize the highly expressed markers on the surface of GSCs, such as CD133, CD44 and integrin, and then guide the nano carriers to accurately target GSCs. Previous studies have shown that nanoparticles coupled with CD133 ligand can significantly improve the targeting and cytotoxicity of lung cancer stem cells, which provides an important reference for the application of this strategy in the targeted therapy of GSCs (Nie et al., 2024). With the over expression characteristics of GSCs surface markers, nano carriers can realize the precise delivery of therapeutic load, improve the therapeutic effect and minimize the Miss target effect and systemic toxicity. In addition, the nano carrier can also be designed to respond to the specific signals of the tumor microenvironment, such as pH value, redox state or enzyme activity changes, so as to specifically trigger the drug release in the local tumor and further improve the accuracy of treatment (Peng et al., 2025).

The integration of GSCs markers into the design of nanocarrier can not only enhance the targeting specificity, but also realize the combined treatment for the inherent multi drug resistance mechanism of glioblastoma GSCs. For example, the construction of a nano platform that can simultaneously deliver chemotherapeutic drugs and GSCs' characteristics related signal pathway inhibitors can effectively enhance the therapeutic effect, and is expected to overcome the strong chemical resistance and tumorigenic potential of GSCs (Zhou et al., 2026). In addition, by modifying tumor microenvironment related molecules, such as loading drugs to alleviate tumor hypoxia, the functionalization of nano carriers can be further realized, providing additional support for improving the therapeutic effect (Tang et al., 2021; Wu J. et al., 2022).

In recent years, the integrated application of biological nano vesicles has become a research hotspot in this field, and the extracellular vesicles derived from stem cells or milk show good application prospects. These vesicles can be engineered to carry siRNA or other nucleic acid therapeutic drugs targeting specific genes of GSCs. With their excellent biocompatibility and internal targeting ability, they can effectively inhibit the activation of carcinogenic pathways in cancer stem cells and reduce tumor proliferation (Ishiguro et al., 2020). Compared with synthetic nanocarriers, these natural nanocarriers can significantly reduce the immunogenicity, which lays a good foundation for the clinical transformation of GSCs targeted therapy.

**FIGURE 6:**
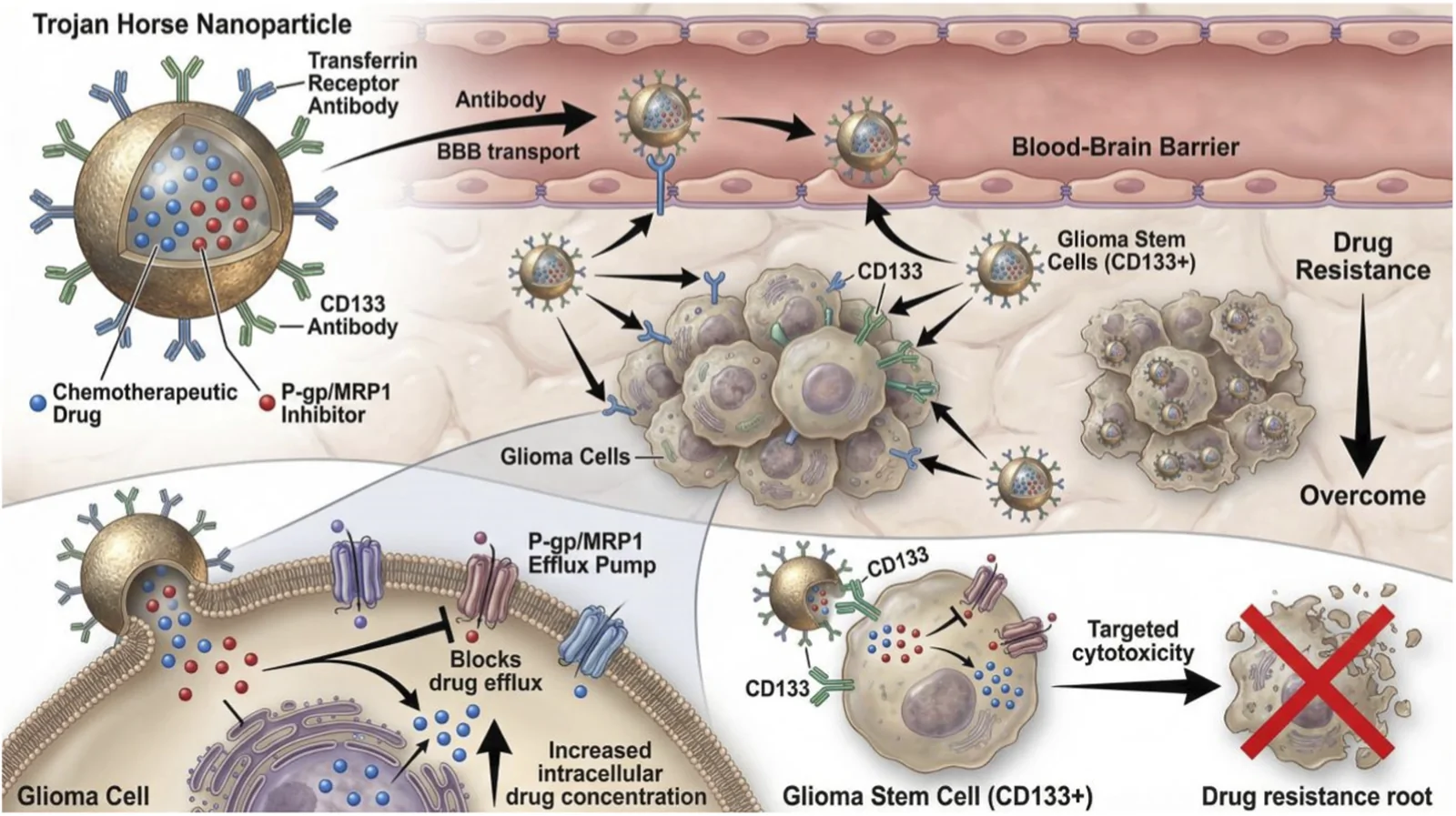
Dual-targeted Trojan horse nanoparticles deliver chemotherapeutic drugs and efflux pump inhibitors across the blood-brain barrier, targeting CD133+ glioma stem cells to reverse chemoresistance.

The combination of nanotechnology and stem cell delivery system provides another efficient strategy for targeted therapy of GSCs. Mesenchymal stem cells or neural stem cells have natural tumor homing properties and can be used as carriers of nanoparticles to enhance their selective accumulation in glioma. This cell nano carrier hybrid strategy can not only improve the drug bioavailability, prolong the circulation time in the body, but also effectively avoid the clearance of the immune system and further improve the treatment efficiency (Yang J. et al., 2024; Liu et al., 2023). However, at present, the strategy still faces many challenges, such as the optimization of drug loading capacity of nanoparticles, the precise control of drug release kinetics, and the security and stability of such complex systems *in vivo*, which still need to be further studied and solved (Figure 6).

In conclusion, the application of nanotechnology in targeted therapy of GSCs has achieved the organic combination of molecular accuracy and innovative drug delivery, which is the frontier strategy in the field of glioma treatment. By integrating GSCs specific markers into the design of nano carriers and making full use of the unique characteristics of tumor microenvironment, such methods are expected to effectively reduce tumor recurrence and improve the survival outcomes of glioma patients. Future research should focus on the optimization of nano platform, the validation of clinical related models, and the resolution of transformation barriers, so as to give full play to its application potential in personalized glioma treatment.

## 9 Conclusion

The core mechanism underlying multidrug resistance in gliomas does not stem from the independent action of a single molecule or pathway; rather, it is a closed-loop regulatory system constructed through the abnormal activation of the Hippo signaling pathway, disordered expression of non-coding RNAs, and mitochondrial dysfunction. In fact, this core loop can also interact with biological processes such as the abnormal drug efflux mediated by MDR transporters, the persistence of stem-like properties in glioma stem cells, and the remodeling of the tumor hypoxic microenvironment, collectively driving the stable formation of drug-resistant phenotypes.

Can the hypoxic microenvironment serve as the initiating trigger for the entire drug resistance regulatory network? Current research evidence sufficiently supports this hypothesis. Tumor hypoxia significantly induces the aberrant expression of key non-coding RNAs, such as miR-210, which directly interfere with mitochondrial metabolism, reshape cellular energy metabolism patterns, and regulate the generation and accumulation of intracellular reactive oxygen species (ROS). The substantial accumulation of mitochondrial ROS activates the RhoA protein through oxidative modification, subsequently triggering the downstream YAP/TAZ signaling axis. Interestingly, activated YAP/TAZ does not merely mediate a unidirectional pathway response; instead, it reversely regulates the transcriptional expression of genes related to mitochondrial function, thereby establishing a self-activating and sustained positive feedback loop for drug resistance.

In most instances, previous studies have treated various inducers of glioma drug resistance as independent and parallel factors. The regulatory model discussed above may disrupt this fragmented understanding by integrating the dispersed drug resistance mechanisms into an interconnected, dynamically balanced network regulatory system, providing a more robust theoretical foundation for the rational design of subsequent combination therapy strategies. This complex regulatory network forms a highly adaptive tumor ecosystem, representing a core challenge in current clinical treatment.

In recent years, the mechanism research has made key progress. Among them, Hippo signaling pathway has been proved to be the key node to regulate glioma proliferation, stem maintenance and immunosuppression; MiRNA, *lncRNA* and *circRNA* have become potential therapeutic targets because of their regulation specificity; The strategies of targeting MDR transporters, intervening stem cell pathway and remodeling microenvironment also provide multiple ideas for reversing drug resistance.

At the application level, the development of nanotechnology provides an important platform for breaking through the bottleneck of blood-brain barrier and achieving accurate drug delivery. Studies have shown that the rational design of nano carriers can realize the collaborative delivery of chemotherapeutic drugs, drug resistance inhibitors and gene therapy agents, and significantly improve the brain bioavailability. When nanotechnology is combined with MDR inhibition, hyperthermia or targeted stem cell strategy, a multimodal treatment system can be constructed to provide an effective path to overcome drug resistance.

In general, the drug-resistant treatment of glioma needs to integrate the overall strategy of “mechanism targeting, microenvironment regulation and precise delivery.” However, the current research still faces many transformation bottlenecks, including the obstacles of *in vivo* delivery of non coding RNA, the optimization of drug loading and release kinetics of nano platforms, the individual differences caused by the heterogeneity of tumor stem cells, and the lack of reliable efficacy monitoring markers.

To implement the aforementioned integration strategy, it is imperative to leverage emerging technologies to drive the transformation of research paradigms from “mechanism description” to “intervention target discovery” and “dynamic and precise decision-making.” Technological innovation serves as the linchpin in breaking through the core barriers that hinder the translation of glioma drug resistance mechanisms from molecular insights to clinical interventions, and in overcoming treatment bottlenecks.

Single-cell sequencing enables the analysis of transcriptional heterogeneity among glioma stem cells, tumor-associated macrophages, and other cellular subsets within the drug-resistant microenvironment. It facilitates the identification of differential activation patterns of drug-resistant pathways across distinct cell lineages, providing a single-cell resolution basis for multi-cell targeted interventions. Spatial transcriptomics further addresses the limitations of traditional sequencing by incorporating spatial dimensions, enabling precise localization of drug resistance molecular markers such as Hippo pathway activity and hypoxia marker expression to specific niches within the tumor microenvironment (Casati et al., 2021). Indeed, significant spatial heterogeneity exists in tumor drug resistance, which correlates with variations in drug penetration gradients and immune cell distribution, thereby elucidating the intrinsic link between microenvironmental spatial architecture and chemotherapy resistance.

Multi-omics integrative analysis effectively overcomes the limitations of relying solely on single molecular markers for drug resistance prediction, compensating for the inadequacy of single-omics approaches in comprehensively capturing the multiple aberrant features of tumor genomes, epigenomes, transcriptomes, and metabolomes. Can the methylation status of the MGMT promoter, the expression of the RAD51AP1-mediated DNA damage repair pathway, and LDHA-related lactate metabolism characteristics be integrated into a model to accurately distinguish between primary and secondary temozolomide-resistant patient subgroups? Previous multi-omics studies utilizing CUT&Tag, SLAM-seq, and RNA-seq confirmed that H3K9 lactylation modification mediates temozolomide resistance by activating LUC7L2 transcription. This finding provides direct experimental evidence supporting the inclusion of lactate metabolism characteristics in multi-omics joint models to enhance the precision of drug resistance subtype stratification (You et al., 2020).

Artificial intelligence and machine learning offer efficient tools for in-depth mining of high-dimensional omics and imaging data. Traditional experimental methods struggle to capture nonlinear correlations between data points, whereas AI can noninvasively predict drug-resistant gene expression profiles based on pathological imaging features, circumventing the invasiveness and delay associated with tissue biopsies. Concurrently, machine learning can model the complex relationships between drug sensitivity and multi-omics characteristics, facilitating the rapid identification of candidate targets for drug resistance reversal, such as YAP/TAZ inhibitors and non-coding RNA-targeted drugs. This closed-loop approach integrating computational and experimental methods significantly reduces the trial-and-error costs of drug screening and accelerates the preclinical development cycle of drug resistance reversal agents (Mo et al., 2022).

The practical application of these cutting-edge technologies must be closely integrated with clinical trials. By synchronously collecting tumor multi-omics data and dynamic liquid biopsy samples during clinical trials, we can continuously monitor the dynamic molecular characteristics of tumor progression and drug resistance evolution. The establishment of a closed-loop system encompassing “drug resistance monitoring, molecular typing, and treatment optimization” based on real-time clinical data is progressively bridging the gap between precision medicine and clinical practice, thereby advancing the clinical implementation of individualized drug resistance interventions for glioma.

Looking ahead, achieving breakthroughs in glioma drug resistance research necessitates a paradigm shift from “mechanism description” to “clinical intervention.” Given the current challenges, the following areas will be the focal points of future research:

Firstly, the precision of preclinical models and the intelligence of delivery strategies must advance in tandem. The current evaluation system, which relies on commercial cell lines and immunodeficient nude mice, often overestimates the efficacy of nanocarriers due to the absence of a complete immune context, failing to accurately reflect drug metabolism and response in an immunocompetent microenvironment. In the future, patient-derived glioma cells (PDCs) and humanized immune reconstitution models should be widely adopted for validation. Moreover, hypoxia, a core feature of the tumor microenvironment, should not merely be viewed as a passive contributor to drug resistance but can be harnessed as an active delivery control switch. Designing hypoxia-responsive nanocarriers (incorporating elements such as azobenzene or nitroimidazole) enables targeted release of therapeutic molecules in hypoxic regions. However, the primary challenge lies in the spatiotemporal heterogeneity of hypoxia signals and the diffusion limitations imposed by high tumor interstitial pressure. Therefore, developing multi-level responsive (pH/enzyme/oxygen partial pressure-linked) and actively permeable platforms is essential to overcome these barriers.

Secondly, equal attention should be given to the immune compatibility and microenvironment remodeling capabilities of nanocarriers. The surface charge, hydrophobicity, and particle size of nanoparticles not only influence their blood-brain barrier penetration efficiency but may also directly induce tumor-associated macrophages to polarize toward the M2 phenotype, exacerbating immunosuppression and accelerating clearance by the reticuloendothelial system. Thus, future carrier designs should prioritize “immune inertness” or “immune modulation” as core parameters, achieving immune evasion through biomimetic membrane coating or CD47 modification. Additionally, targeting neutrophil polarization—an emerging intervention point (where the N2 tumor-promoting phenotype impedes drug penetration via physical barriers and inflammatory mediators)—by developing auxiliary strategies that modulate its polarization pathways holds promise for enhancing the synergistic effects of chemotherapy and immunotherapy.

Thirdly, drug delivery should evolve from “physical transport” to “biological decision-making,” integrating tumor stress response mechanisms. Future intelligent drug delivery systems should incorporate microenvironment-sensing modules to enable “on-demand” release of therapeutic molecules based on local signals such as ATP, lactic acid, or specific enzyme activities. Simultaneously, incorporating tumor stress responses (e.g., endoplasmic reticulum stress, oxidative stress) into delivery designs, utilizing stress-induced specific promoters or sensitive elements, can further enhance the lesion-specific release of drugs. Preclinical data indicate that delivery systems integrating stress response mechanisms outperform traditional formulations in both anti-tumor activity and tolerability, suggesting this strategy could significantly improve clinical translation success rates.

Fourthly, the advent of gene editing technologies offers a radical approach to reversing drug resistance. Unlike traditional methods that inhibit drug efflux or block signaling pathways, the CRISPR-Cas9 system can permanently knock out drug resistance-driving genes (e.g., MGMT, YAP, or ABCB1) at the genomic level or correct mutations through base editing, fundamentally undermining tumor drug resistance. When combined with the aforementioned nanodelivery platforms (e.g., lipid nanoparticles or exosomes) for precise *in vivo* delivery, CRISPR tools may break the current cycle of “targeting-resistance-retargeting.” Ensuring editing efficiency and safety while avoiding off-target effects remains the primary challenge for clinical translation of this strategy.

In conclusion, the ultimate conquest of glioma drug resistance hinges on the precision of model systems, the intelligence of delivery vectors, the decision-making capacity of treatment strategies, and the radical intervention of gene editing—these four pillars must mutually reinforce each other to propel glioma treatment from a “passive response to drug resistance” to a new era of “proactive solution design,” ultimately delivering substantial survival benefits to patients.
